# NeuroConstruct-based implementation of structured-light stimulated retinal circuitry

**DOI:** 10.1186/s12868-020-00578-0

**Published:** 2020-06-24

**Authors:** Miriam Elbaz, Rachel Buterman, Elishai Ezra Tsur

**Affiliations:** 1 Jerusalem College of Technology, Jerusalem, Israel; 2 Neuro-Biomorphic Engineering Lab, Department of Mathematics and Computer Science, Open University of Israel, Raanana, Israel

**Keywords:** Neuron, Computational neuroscience, Neuronal modeling, NeuroML

## Abstract

**Background:**

Retinal circuitry provides a fundamental window to neural networks, featuring widely investigated visual phenomena ranging from direction selectivity to fast detection of approaching motion. As the divide between experimental and theoretical visual neuroscience is fading, neuronal modeling has proven to be important for retinal research. In neuronal modeling a delicate balance is maintained between bio-plausibility and model tractability, giving rise to myriad modeling frameworks. One biologically detailed framework for neuro modeling is NeuroConstruct, which facilitates the creation, visualization and analysis of neural networks in 3D.

**Results:**

Here, we extended NeuroConstruct to support the generation of structured visual stimuli, to feature different synaptic dynamics, to allow for heterogeneous synapse distribution and to enable rule-based synaptic connectivity between cell populations. We utilized this framework to demonstrate a simulation of a dense plexus of biologically realistic and morphologically detailed starburst amacrine cells. The amacrine cells were connected to a ganglion cell and stimulated with expanding and collapsing rings of light.

**Conclusions:**

This framework provides a powerful toolset for the investigation of the yet elusive underlying mechanisms of retinal computations such as direction selectivity. Particularly, we showcased the way NeuroConstruct can be extended to support advanced field-specific neuro-modeling.

## Background

Computational modeling of neuronal dynamics is paramount for neuroscientific research. Particularly, conductance-based modeling of the neural network holds a promise to uncover biological mechanisms which underlie higher functional behavior of neuronal frameworks [1, 2]. Neuronal modeling is often comprised of multiple specification layers, including morphological description, biophysical characterization, cell positioning, connectivity schemes, synapse definition and stimuli depiction. These models often incorporate detailed experimentally reconstructed 3D morphologies of neurons. Integrating all of the above into one parameterized model is a challenge many computational neuroscientists face, particularly in the face of the great availability of modeling environments [3].

To mitigate this challenge, Gleeson and colleagues developed NeuroConstruct, which facilitates the creation, visualization, and analysis of neural networks in 3D [4]. Each neuron is defined as a multi-compartmentalized entity, represented by an equivalent circuit and connecting with other neurons via software-defined point processes–synapses. NeuroConstruct was implemented to support different numerical solvers such as GENESIS and NEURON. Importantly, it uses the latest NeuroML specifications [5]. NeuroML, also developed by Gleeson and colleagues, is a specification language based on the Extensible Markup Language (XML). It aims at standardizing the definition of detailed neuronal models.

Over the past decade, NeuroConstruct was widely adopted throughout the scientific community. For example, it was utilized by Rothman and colleagues to investigate a detailed layer 5 pyramidal cell model with dendritically distributed excitatory and inhibitory synaptic input [6]. Vervaeke and colleagues also used NeuroConstruct to create an electrically coupled cerebellar Golgi cell network to explain the spread of desynchronization in this network following sparse synaptic activation [7]. Lastly, Hanson and colleagues used NeuroConstruct to investigate how synaptic pathologies can underlie cognitive impairments [8].

Retinal circuitry provides a fundamental window to neural networks [9] and among its major responsibilities are: (1) Detection of dim light flashes–even at the level of single photons; (2) High sensitivity to changes in textures, enabling the detection of moving patterns of light despite a constant level of illumination; (3) Detection of differential motion, enabling the differentiation of global motion (all elements in the Field Of View (FOV) are moving together) and local motion (one object within the FOV is moving); (4) Fast detection of approaching objects; (5) Encoding of spatial structures with spike latencies (cells in dark regions respond faster than cells in brighter regions). Each of these fundamental phenomena is widely investigated by the research community, as some of the neuronal mechanisms are elusive. One of the classic open questions in retinal circuitry is the underlying mechanism of directional sensitivity: the sensitivity of Direction Selective Ganglion Cells (DSGC) to a moving visual stimulus in one preferred direction. This fascinating phenomenon represents the ability of the retina to discard information and focus on one aspect of the visual scene. A detailed review of the above is given by Gollisch and colleagues [10]. These phenomena are commonly experimentally addressed with the projection of structured light (e.g. drifting bars, rings of light, etc.) on a retina [16]. Modeled virtual stimulation of structured light is therefore essential for model validation using experimentally derived data as well as for the computational elucidation of the underlaying mechanisms.

While proved useful, NeuroConstruct is often extended to support different neuronal architectures. The neural networks of the retina are comprised of a multitude of neuron types and organized within a layered structure. Each network has a distinct function in transferring visual information [11]. One of the most investigated subjects in the retina is the interface between Starburst Amacrine Cells (SACs) and DSGCs. SACs differentially inhibit DSGCs in a phenomenon that underlies their receptive field properties, giving rise to their fundamental properties—being sensitive to a stimulus moving in a particular direction. SACs are predominantly driven by Bipolar Cells (BCs). BCs receive inputs from a set of light-sensitive photoreceptor cells and mainly respond with sustained graded potentials. The SACs—BCs interface is comprised of different synapses which exhibit diverse dynamics. These synapses are not uniformly distributed and have distinct connectivity schemes. Adjacent SACs also reciprocally inhibit each other, giving rise to intricate properties which were shown to strengthen the directionality of DSGCs. An elaborate discussion is given in [12]. Modeling such a network requires the support of unique synaptic patterns as well as the careful implementation of the visual stimuli. NeuroConstruct, which natively does not support rule-based feature distribution and dynamics, must therefore be extended in order to support retinal circuitry modeling.

In this work, we extended NeuroConstruct to support retinal circuitry with some of its specific characteristics, connectivity patterns, and structured visual stimulation. We exemplified this framework using a simulation of two overlying grids of biologically realistic, morphologically detailed SACs, which were connected to a ganglion cell and stimulated with expanding and collapsing rings of light. This extended framework can be potentially utilized to investigate the elusive underlying mechanism of direction selectivity, as well as other retinal phenomena such as ganglion cell sensitivity to moving texture [13], differential motion [14], and approaching motion [15].

## Implementation

### Framework

**Fig. 1:**
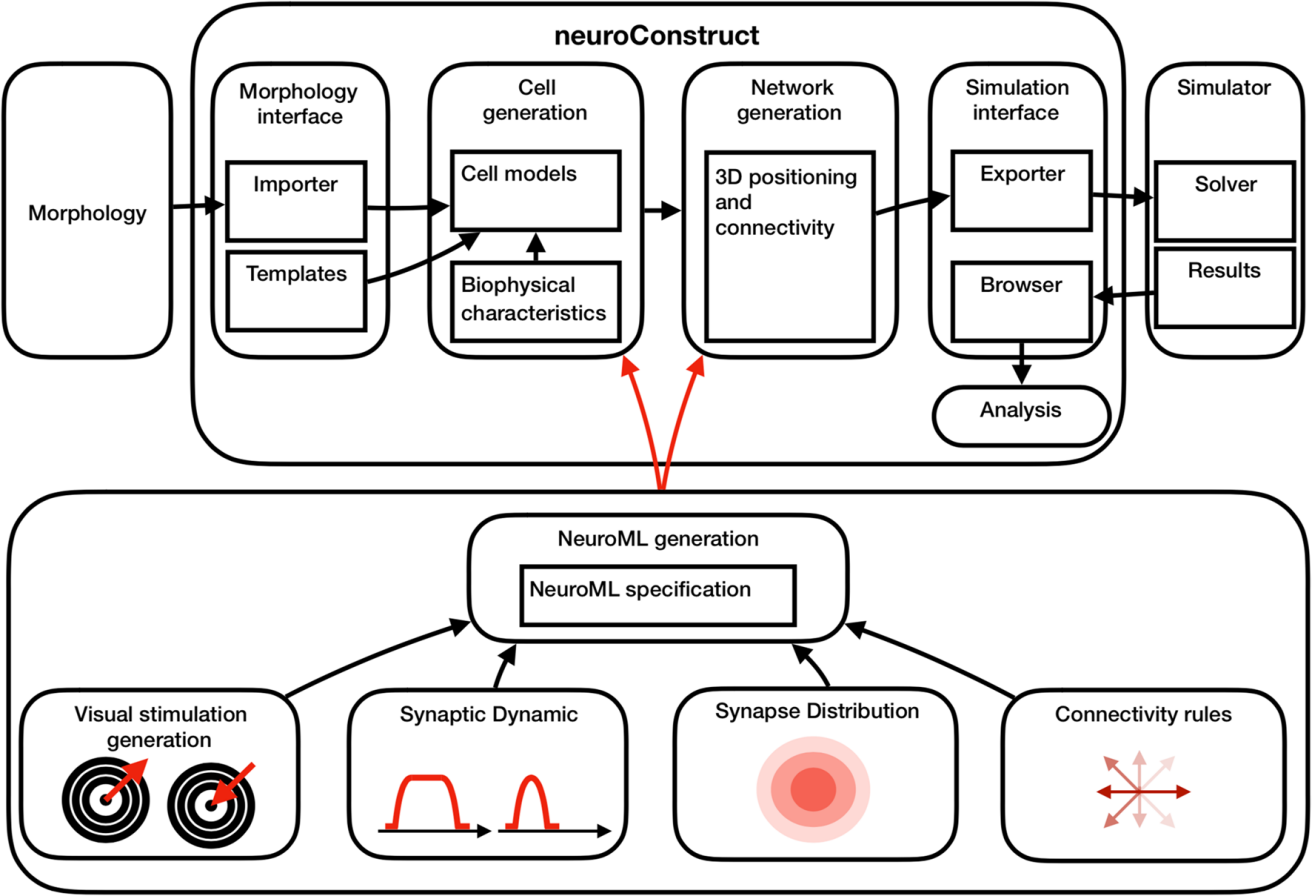
*Framework schematic*. The NeuroConstruct framework is comprised of the morphology import, cell generation, network generation, simulation interface, and data export module. Our extensions support the generation of visual stimuli, heterogenous dynamic properties of synapses, synapse distribution and connectivity rules. Our extension modules interface with NeuroConstruct via a NeuroML compiler

A schematic of the framework is shown in Fig. 1. The NeuroConstruct framework is comprised of five main components: (1) The morphology interface, with which biologically realistic NeuroML-specified reconstructed models of neuronal morphologies are imported, as well as existing morphological templates aimed at generating simple neuronal models with few compartments for rapid hypothesis testing; (2) Cell generation module, in which neurons are encapsulated as groups of sections, distinguished as axons, somata, and dendrites. Compartments are biophysically defined with a set of channel mechanisms. These mechanisms (e.g. membrane capacitance, axial resistance and activity dependent intracellular Ca^2+^ concentrations) are specified using the NeuroML framework and can be distributed throughout the sections. Notably, mechanisms can be specified manually or utilized from an existing template; (3) A network generation engine, in which neurons are arranged in cell groups (according to their specified types) and placed in 3D individually or according to a predefined pattern (such as evenly spaced packing or randomized placement). Following placement, synaptic connections are defined within or between cell groups. Traditionally, synaptic connections are defined in NeuroConstruct using connectivity patterns, which can be morphologically (proximity of pre- and post-synaptic sections) or volume based. Both can be parameterized (number of synapses per cells, connection lengths) to allow control of synaptic distribution. In this component, the stimuli of the network are defined as external activation, traditionally as either fixed current steps or random spike trains of synaptic input; (4) Simulation interface, in which the simulation data is organized and formulated to run on numerical solvers such as NEURON or GENESIS; (5) Once the solver retrieves the simulation results, data can be browsed and analyzed within NeuroConstruct, as well as exported to MATLAB or EXCEL.

NeuroConstruct provides a powerful tool for neuronal modeling. We enhance it further with a series of scripts and packages to enable an increased level of details, specifically tailored to parts of the retinal circuitry. The following details must be addressed in order to numerically investigate retinal circuitry: (1) Generation of structured visual stimuli. Over the past decades, several visual stimuli were used to investigate the different aspects of visual processing. For example, moving bars of light, as well as expanding and collapsing rings of light, were used to investigate motion processing, particularly directional selectivity [16]. However, the generation of visual stimuli in a simulation environment is far from trivial, as the stimulation for every synapse in the model has to be resolved for every time interval according to the chosen light pattern; (2) The dynamic of the synaptic response may differ according to its distance from the cell soma. Most notably, neuronal activity can be characterized as sustained or transient, where the neural response persists during the duration of the stimuli, or only during the onset/offset of the stimulus [17]; (3) Synapse distribution may not be homogeneous across the neuronal dendritic tree, particularly, synapse density may depend upon its distance from the cell soma [18]; (4) Rule-based synaptic connectivity may include intricate considerations such as the orientation of the section relative to the soma. Extending NeuroConstruct regarding the aforementioned aspects is an important steppingstone in the adoption of the framework to model retina circuitry for visual processing.

The four extensions proposed above were implemented with Python and coupled with a new compiler which converts user code to NeuroML specifications. These specification files can be incorporated into NeuroConstruct for visualization, simulation and analysis. Description and installation instructions are given in the Additional files. Code examples (python) are given as Additional file 1 and described below.

### Results

**Fig. 2:**
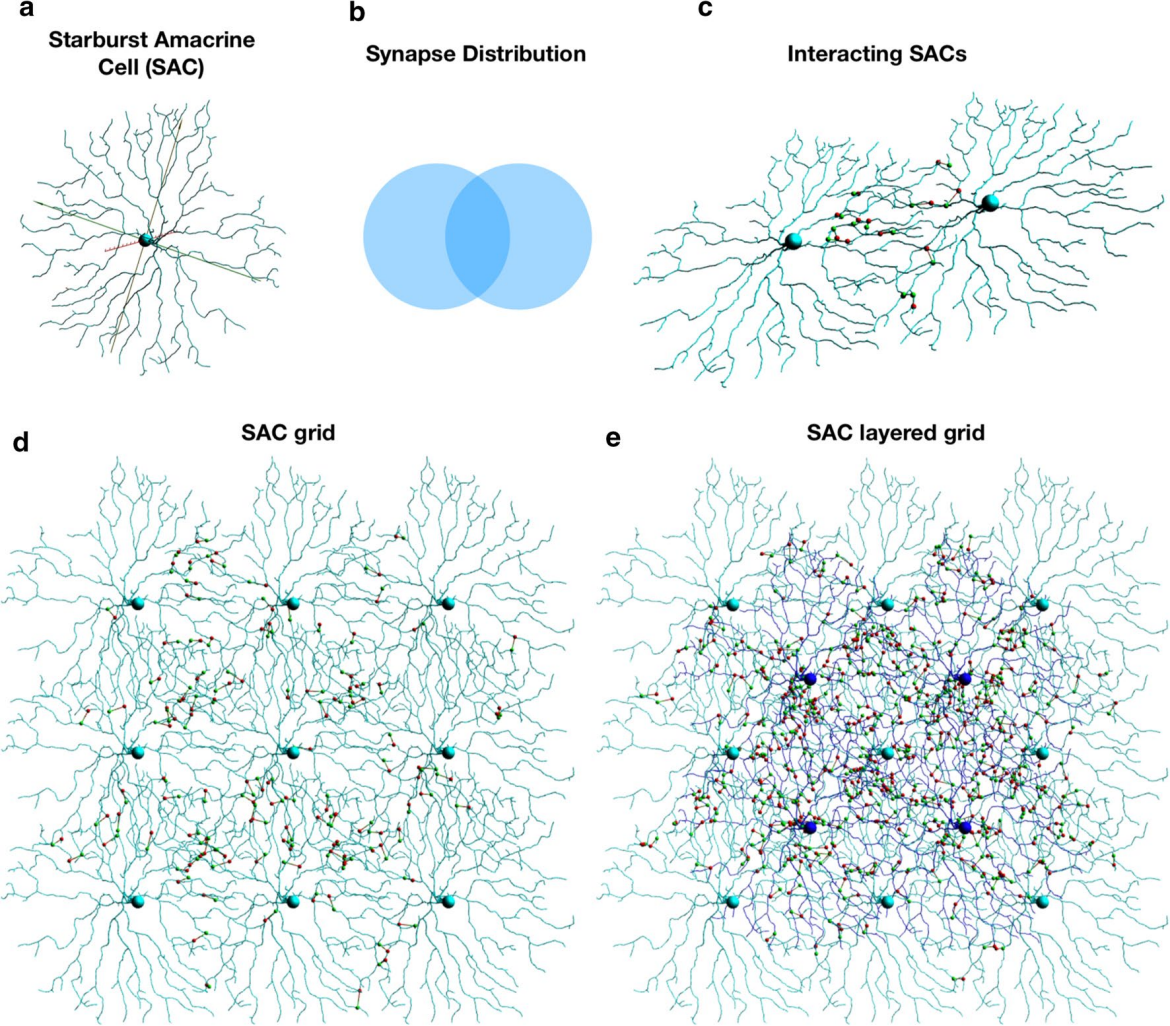
SAC plexus. **a** Biologically realistic morphology of a SAC. **b** Schematic of inter-SAC inhibitory synaptic signaling distribution. Synapses are restricted to distal 1/3 of the SAC’s dendritic tree. **c** Two intersecting SAC’s and the formed inhibitory synapses. **d** a 3x3 grid of intersecting SACs and their synapses. **e** the SAC plexus created with 2 overlapping 3x3 and 2x2 grids of biologically realistic SACs

Here we show the utilization of NeuroConstruct and our extension modules for modeling the SAC plexus in the retina. SACs are predominantly stimulated by BCs and provide inhibitory signals to ganglion cells, which convey the visual stimuli through the optic nerve to the brain. SACs are essential for efficient direction selectivity via several mechanisms [19]. SACs are radially symmetric retinal interneurons, with a synonymous axon/dendrite functionality, termed processes (Fig. 2a). They tile the retina in an extensive overlapping pattern (Fig. 2b), as adjacent SACs reciprocally inhibit each other via GABA junctions. Notably, these inhibitory synapses are restricted to the distal 1/3 of the dendritic tree [18] (Fig. 2c). In our model, we utilized NeuroML specification to define a 3 × 3 grid of SACs (Fig. 2d), then overlaid it with a second 2x2 grid of SACs to achieve dense coverage (Fig. 2e). SACs plexus structure was inspired by [16]. We hypothesized that two overlapping grids of SACs will be sufficient to demonstrate direction selectivity. SAC morphology was adopted from [18], and available via Neuromorpho.org (ID: NMO_50993). See the Additional file 2 for example implementation.

**Fig. 3:**
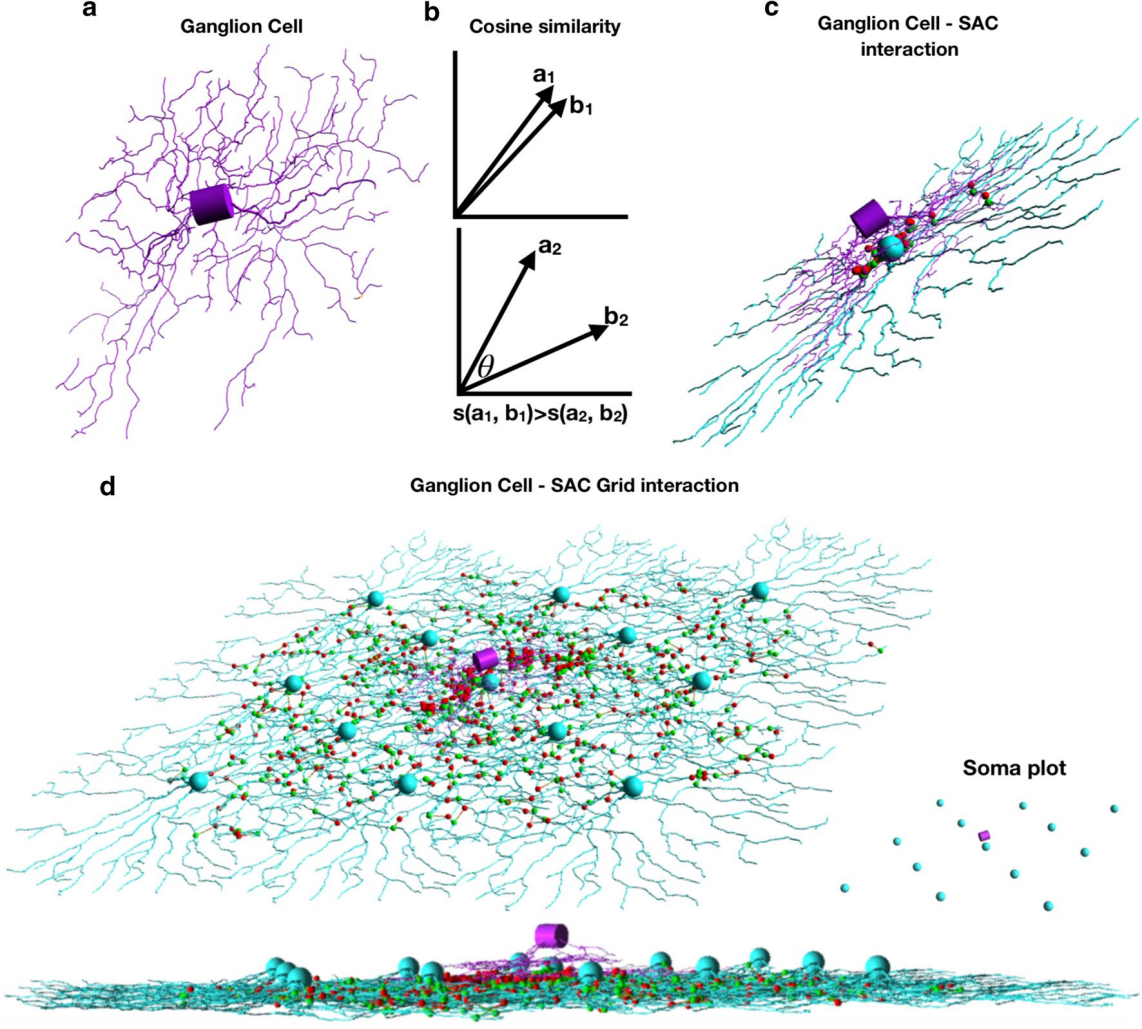
SAC—DSGC selective synaptic connections. **a** Biologically realistic morphology of a DSGC. **b** Schematics of cosine similarity showing higher similarity for the vectors in the top graph, relative to vectors at the bottom graph. **c** Connectivity pattern between biologically realistic SAC and DSGC. **d** Three views of the connectivity pattern between the SAC plexus and the DSGC

One of the mechanisms in which SACs contribute to direction selectivity is based on selective synaptic connections to a DSGC, which has a preferred direction of visual stimulus to which it strongly responds (Fig. 3a). Particularly, SAC processes form GABAergic synaptic connections with DSGC, where the process is oriented in the DSGC’s null direction. To implement this connectivity rule, we defined the probability of a synapse formation as dependent upon the inverse cosine similarity between the section’s directionality (relative to the soma) and a predefined preferred-direction of the DSGC. Illustration of the cosine similarity measure is shown in Fig. 3b and the connection between a SAC and DSGC is shown in Fig. 3c. In our model, we positioned a DSGC in the middle of the SACs layers, slightly vertical above, where it comes into close contact with 5 neighboring SACs, forming synapses where appropriate. Architecture was inspired by the results of [16] (Fig. 3d). See the Additional file 3 for example implementation.

**Fig. 4:**
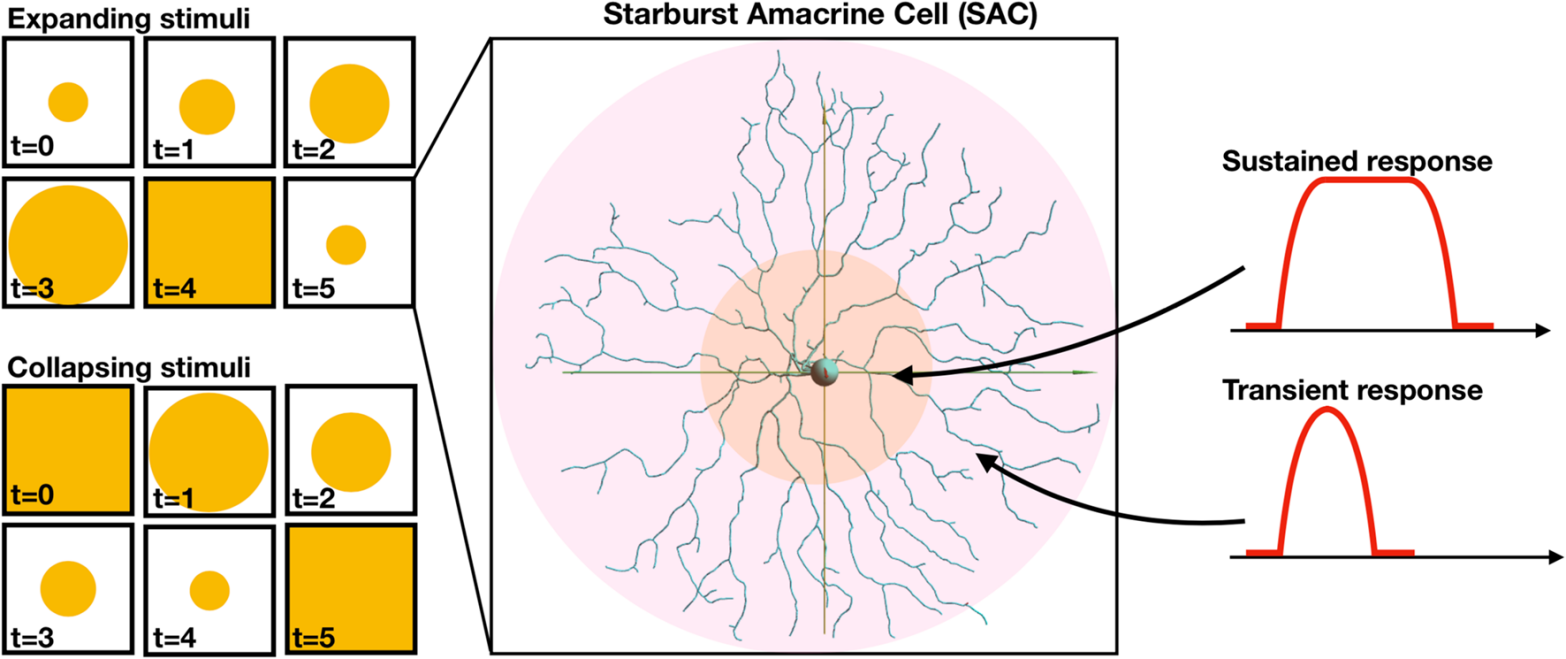
Visual stimuli and dynamic distribution. (LEFT) Schematics of expanding and collapsing rings of lights used for visual stimulation. Time (t) is in arbitrary units. (RIGHT) Schematic of the distribution of the synaptic dynamic properties of the SACs, distinguishing between the inner proximal 1/3 of the dendritic tree and the other 2/3, featuring synapses with sustained and transient response respectively

**Fig. 5:**
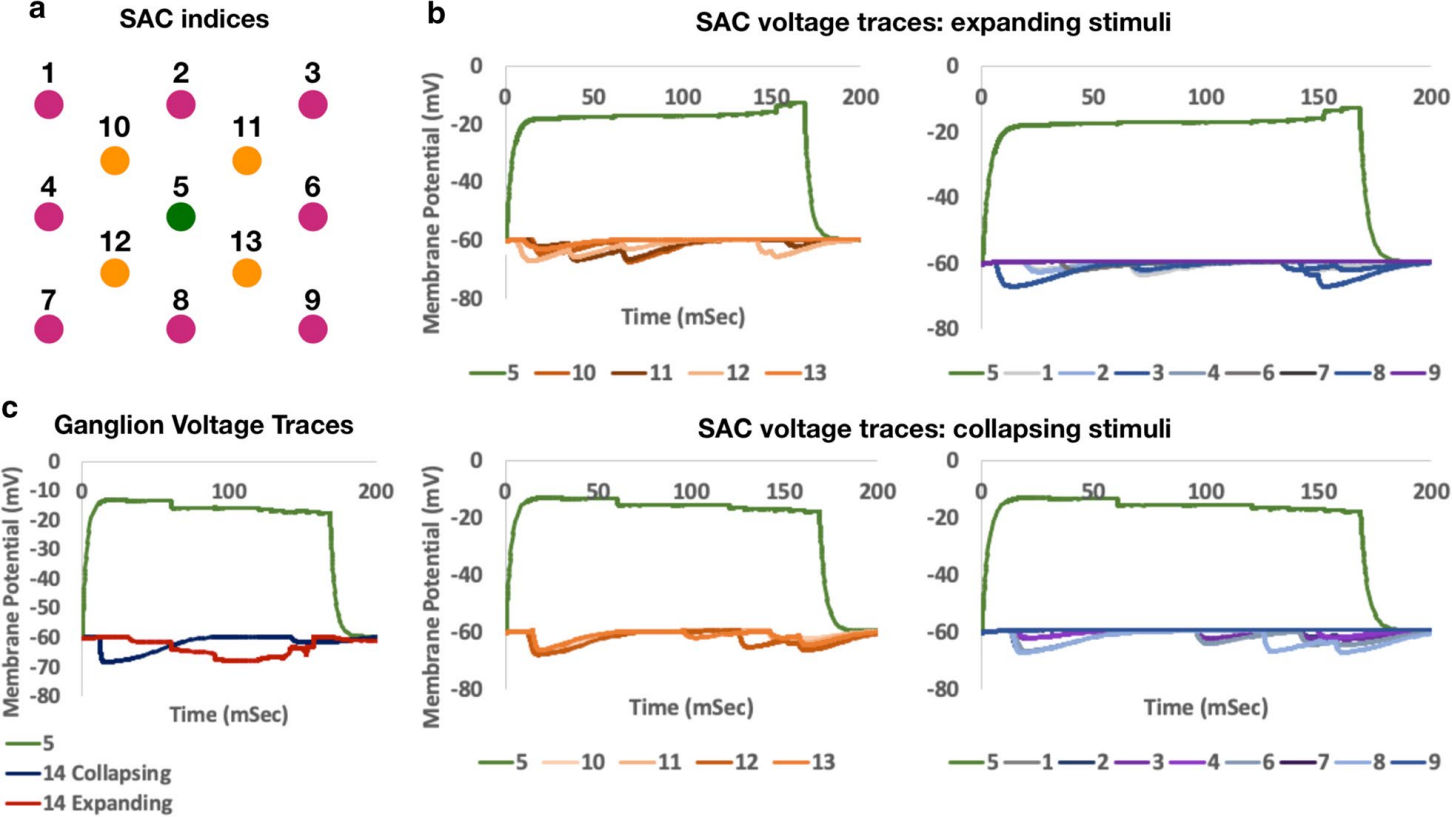
Simulation results. **a** Indices label the Starburst Amacrine Cell (SAC) within the 2 layered plexus. **b** SAC voltage (somata) traces response for expanding (top) and collapsing (bottom) rings of light. Light cycles frequency is 6 Hz (illumination period of 166 mSec). **c** Ganglion voltage (soma) traces (labeled as 14) response for expanding and collapsing rings of light). A voltage trance for the 5th SAC was added as a reference

For a visual stimulus, we wrote a script which specifies expanding and collapsing rings of light and calculates the injected current for each time interval of the simulation (Fig. 4, left panel). Following the model suggested by Greene and colleagues [17], BCs—SACs proximal (1/3) synapses were defined as sustained and others as transient (Fig. 4, right panel). These synapses were distributed according to the synapse distribution model proposed by Vlasits and colleagues [18], where they are apparent in 30% of the processes’ sections at the proximal 1/3 of the dendritic tree, in 3% of the distal 1/3 and linearly distributed in between according to: [0.03(x−60) + 0.3(100−x)]/40, where x is the section’s distance from the soma. Stimuli were applied via BCs solely on the middle SAC (Fig. 5a, indexed 5, colored green), which has a propagating effect on the rest via their synaptic connections. Response dynamics of each synapse is dependent upon the location of the synapse, particularly its distance from the soma.

We execute the simulation over NeuroConstruct (using NEURON API) and our extension library for both expanding and collapsing rings of light. We indexed the cells (Fig. 5a) and traced the voltage at their somata in a constant 25 mSec interval. Results are shown in Fig. 5b. The middle SAC (Fig. 5a, indexed 5, colored green) generated a sustained ~ 40 mV depolarization response throughout the stimulation period. The rest of the SACs, connected to the middle SAC via GABAergic synapses, responded in a ~ 8 mV hyperpolarization response as expected. The monitored voltage waves in the cells throughout the SAC grid are shown in Fig. 5a, where we differentiated the inner cells (indexed 10−13, colored orange) from the peripheral cells (indexed 1–4 and 6–9, colored purple). Note that the only cell which is activated within the SAC grid is the middle cell (indexed 5). The middle cell inhibits his neighboring cells, which in turn inhibit their neighbors. While the response of the activated SAC (indexed 5) at the soma is similar for both expanding and collapsing stimuli, the propagated inhibitory signals, transmitted to the DSGC, have a different net effect on its voltage, depending on the direction of the projected structured light (Fig. 5c). For a structured projection of collapsing rings of light, the DSGC respond with fast hyperpolarization and slow polarization (we hypothesize that this is due the relatively *large* number of SAC at the periphery and the *sustained* dynamics of BC-SAC synapses at the proximal part of the SAC). On the contrast, the DSGC respond to structured projection of expanding rings of the light with gradual hyperpolarization and fast polarization (we hypothesize that this is due to the relatively small number of SAC at the center and the *transient* dynamics of BC-SAC synapses at the proximal part of the SAC). The results clearly demonstrate the directionality of the network, which we hypothesized to have emerged from the network topology and synaptic distribution. See the Additional files 4 and 5 for example implementation of collapsing and expanding stimuli respectively. The scripts referenced throughout this section are utilizing various functions which are given in the Additional file 6.

## Conclusions

Understanding retinal information processing and its underlying neural circuitry has attracted significant attention in the past decades. The traditional view of the retina, as being a relay or a filter for visual information, has been shattered by recent advancements which demonstrate the importance of the retina in solving a diverse set of computational tasks, providing processed data to downstream brain areas [9]. Many of the retinal mechanisms for vision processing are still comprehensively investigated, as their complexity and computational importance become evident.

Models of retinal processing span over a wide spectrum of biological plausibility. Some are highly abstract, neglecting biological details, often relying on the linear–nonlinear (LN) model in which a cascade of linear and non-linear functions is applied over a visual stimulus [20] or on neural coding [21, 22]. Others extend LN with retina-inspired sampling functions, spatiotemporal filtering, and color-coding [23]. Models can also focus on the layered structure of the retina, either by reproducing connectivity in successive retinal layers with detailed cellular and synaptic parameters [24] or by defining a spatiotemporal filter cascade with shunting feedback to represent the process of visual inputs traversing throughout the retina layers [25]. However, these models lack the biological plausibility needed to model retinal processing at the level of molecules and dynamics, which are essential for the support or elimination of biologically detailed hypotheses regarding retinal mechanisms.

Simulating detailed model is not a trivial undertaking. Numerous frameworks must be developed, aiming at increasing modeling productivity and encapsulating technical and tedious processes from neuroscientists, allowing them to focus on the creative parts of their work [26]. One of the most important environments for detailed neuronal simulations is NEURON, which provides a numerical framework for biologically detailed models [27]. However, NEURON was developed with Hoc, a C-inspired Domain Specific programming Language (DSL), which limits NEURON’s utility and maintenance, establishing the need for a friendly yet comprehensive modeling environment.

In an attempt to make software easy to use and develop, Lieberman and colleagues put an emphasis on user intuition in their seminal paper: “End-User Development (EUD): An Emerging Paradigm” [28]. According to the EUD approach, users should focus their attention on the creative process of their work, not worrying about neither syntax nor hardware-related aspects. In the context of neuronal modeling, Gleeson and colleagues developed NeuroConstruct in which models can be defined in 3D (with ideally little need for writing code) and simulated with NEURON [4]. In this work we extended NeuroConstruct in multiple dimensions, allowing scientists to conveniently define visual stimulation and to easily distribute synapses using retinal-relevant connectivity rules and response dynamics. Moreover, we aimed to showcase the ways in which NeuroConstruct can be extended to support advanced field-specific neuromodelling, inspiring neuroscientists to utilize the immense power of NeuroConstruct toward their own neuromodeling exploits.

## Availability and requirements

*Project name* NeuroConstruct-based implementation of Retinal Circuitry.

*Project home page* Code is provided as supplemental information.

*Operating system* Windows.

*Programming language* Python, Java.

*Other requirements* Java 8u201, Notepad 7.6.4, NeuroConstruct 1.7.2, NEURON 7.2, Python 3.7. Please see detailed description and code examples in the supplemental information.

*License* MIT license

*Any restrictions to use by non-academics* None

*Note* The installation instructions of the framework, with code examples, are provided as supplemental information. As described above, the framework uses a series of dependable modules, which are freely accessible and described in the supplemental information.

## Supplementary information

**Additional file 1.** Installation and manual.

**Additional file 2.** Code example 1: Network creation.

**Additional file 3.** Code example 2: Synapse creation and distribution.

**Additional file 4.** Code example 3: Collapsing light stimulation.

**Additional file 5.** Code example 4: Expanding light stimulation.

**Additional file 6.** Code example 4: General functions.

## Data Availability

All data generated or analyzed during this study are included in this published article [and its supplementary information files].

## Abbreviations

BC: Bipolar cell
DSGC: Direction-selective ganglion cell
DSL: Domain specific programming language
EUD: End-user development
XML: Extensible markup language
FOV: Field of view
LN: linear–nonlinear
SAC: Starburst Amacrine cells
