# Supplementary material for: NeuroConstruct-based implementation of structured-light stimulated retinal circuitry
Source: BMC Neurosci. 2020 Jun 24;21:28. doi: 10.1186/s12868-020-00578-0 (PMC7315481; doi:10.1186/s12868-020-00578-0)
Supplement: Supplementary file 1 — Additional file 1. Installation and manual. [file 12868_2020_578_MOESM1_ESM.pdf]

NeuroConstruct-based implementation of Retinal Circuitry

Before initiating a project, you should have several software packages installed, the main one is neuroConstruct. Note: this work with the included software were tested on *windows*

<u>Prerequisite Installations</u>

- ***JAVA:*** neroConstruct was developed with JAVA and it is therefore essential. http://www.oracle.com/technetwork/java/javase/downloads/index.html
  Download the 8u201 version.Make sure you download the JDK package

- **NotePad++**: Notepad++ is very comfortable for viewing and editing scripts: https://notepad-plus-plus.org/download/v7.6.4.htm. Make sure to download the suitable version to your computer

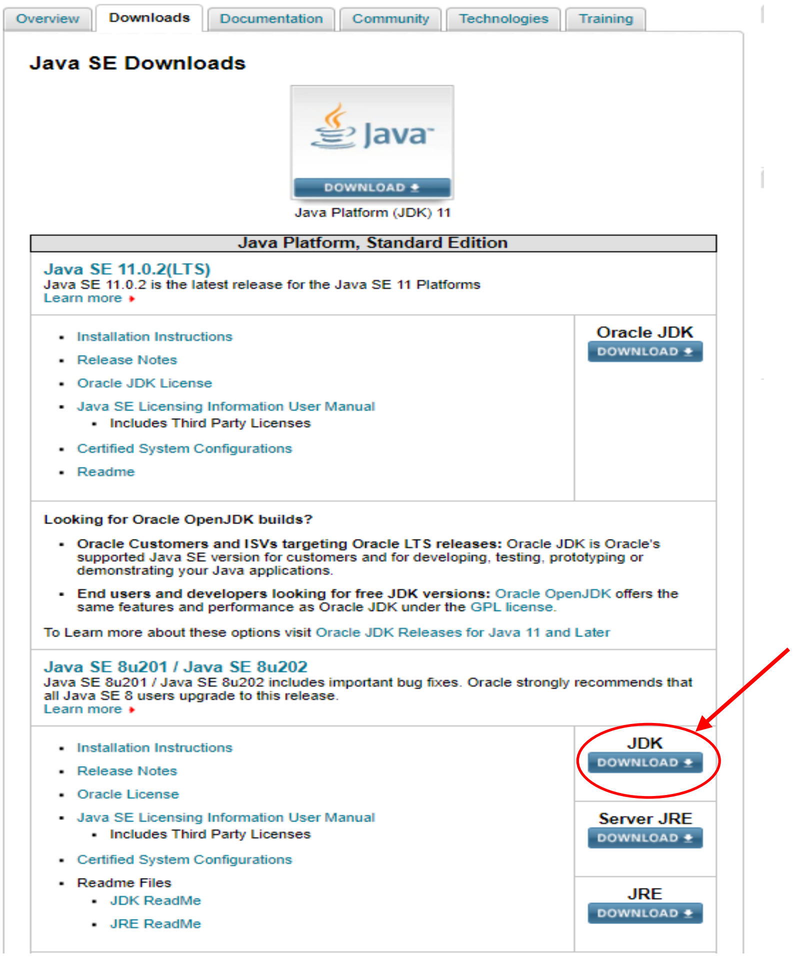

- ***neuroConstruct:*** Follow the installation tutorial of neuroConstruct in: https://github.com/NeuralEnsemble/neuroConstruct/blob/master/INSTALL.md
  make sure to install the binary release (zip file) from: https://github.com/NeuralEnsemble/neuroConstruct/releases.
  This work was tested with version 1.7.2

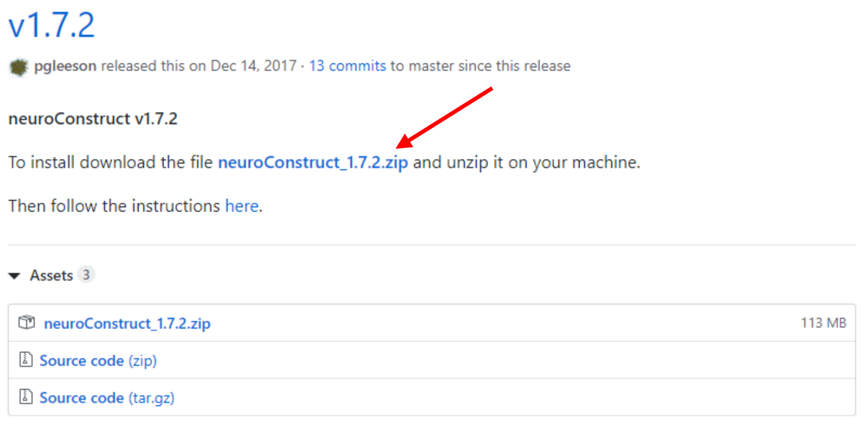

- ***NEURON:***
  Neuron is a simulation environment for modeling individual and networks of neurons.
  Download *NEURON* from: https://neuron.yale.edu/ftp/neuron/versions/
  This work was tested with version 7.2

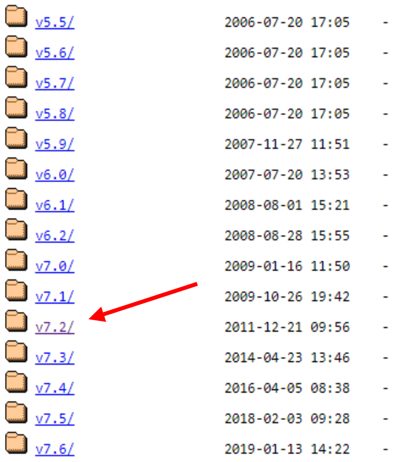

Make sure to work with NEURON v7.2 and neuroConstruct v1.7.2

- ***Anaconda.*** *neuroConstruct scripts are written in Python*. A straight forward way to deploy python is by using *anaconda.* Download anaconda desktop graphical user interface from: https://www.anaconda.com/distribution/#download-section
  This work was tested with python is 3.7

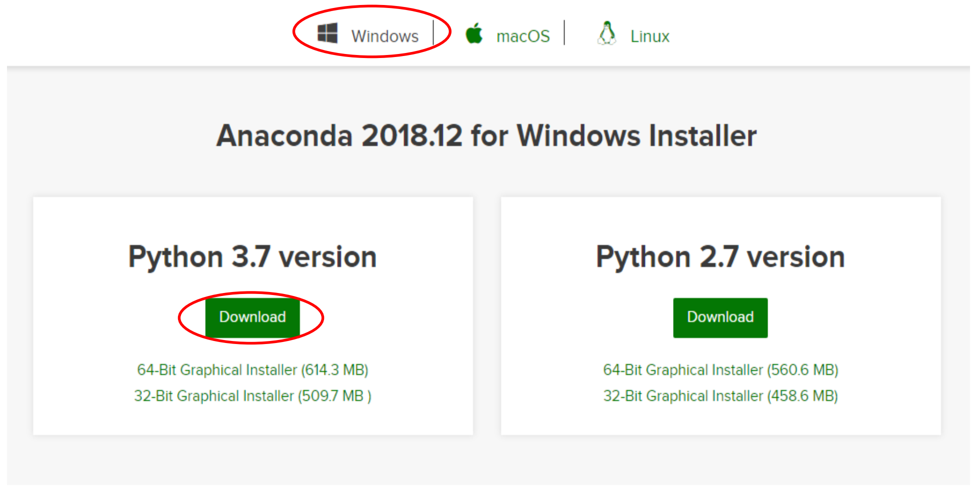

- ***Spyder:*** Spyder is the scientific python development environment. Easiest way to install Spyder is with anaconda navigator (downloaded earlier):

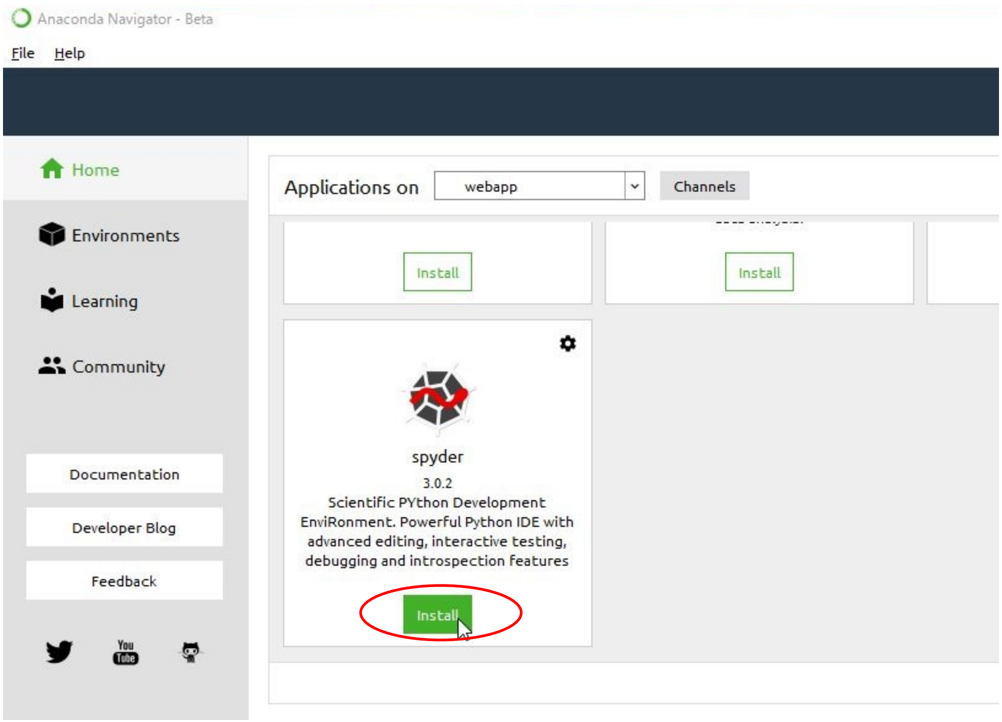

- ***NeuroML.*** XML-based description language that provides a common data format for defining and exchanging descriptions of neuronal cell and network models.
  To download neuroML, open the command line of the *anaconda prompt (downloaded earlier) and* write: *pip install pyneuroml*

<u>Sanity check</u>

Open a project in neuroConstruct using one of their examples. You can find them in your downloaded neuroConstruct package

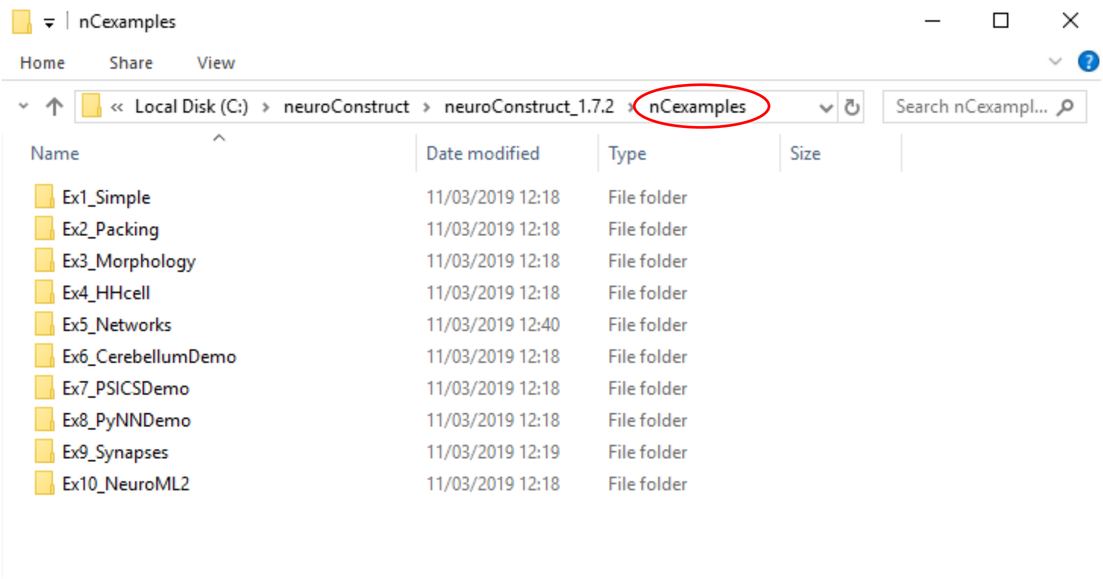

•

NeuroConstruct for retinal circuitry

- **Choose a cell morphology to work on.** We used morphologies from the Neuromorpho data base available in: http://neuromorpho.org/index.jsp
  We used a starburst amacrine cell (from a mouse retina) available in:
  http://neuromorpho.org/neuron_info.jsp?neuron_name=WT1-10-AC

  The downloaded zip file is comprised of an *swc* file, which can be imported to neuroConstruct

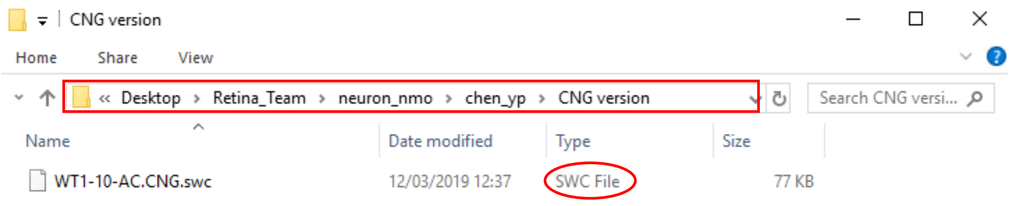

- ***Setting neuroConstruct properties:***
  open neuroConstruct: file-> new project->

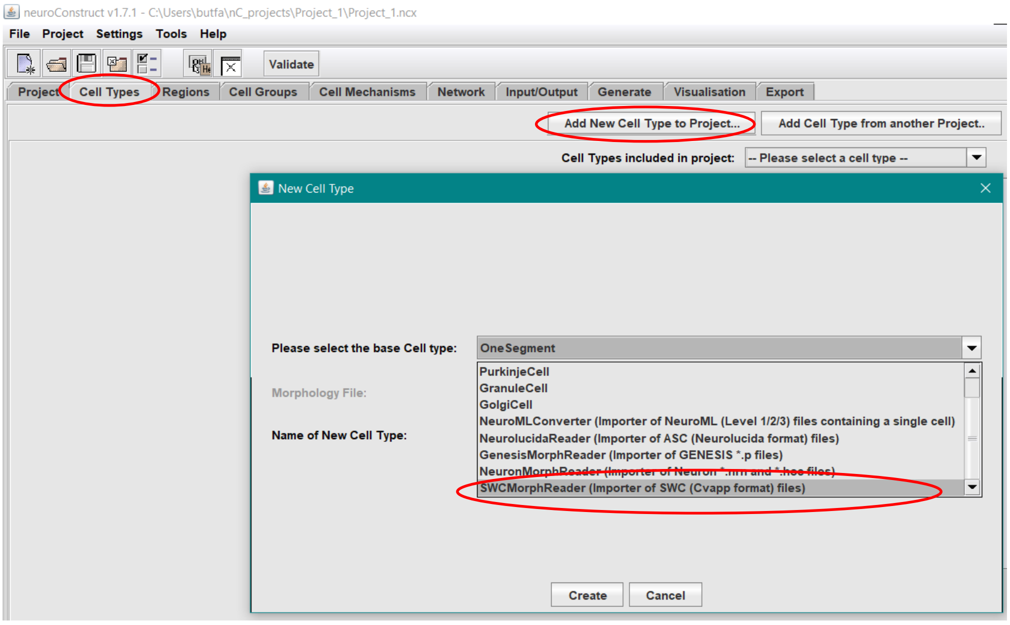

•

After choosing the wanted swc file, you have to add a morphology file which can be found in the same folder where the swc exists.

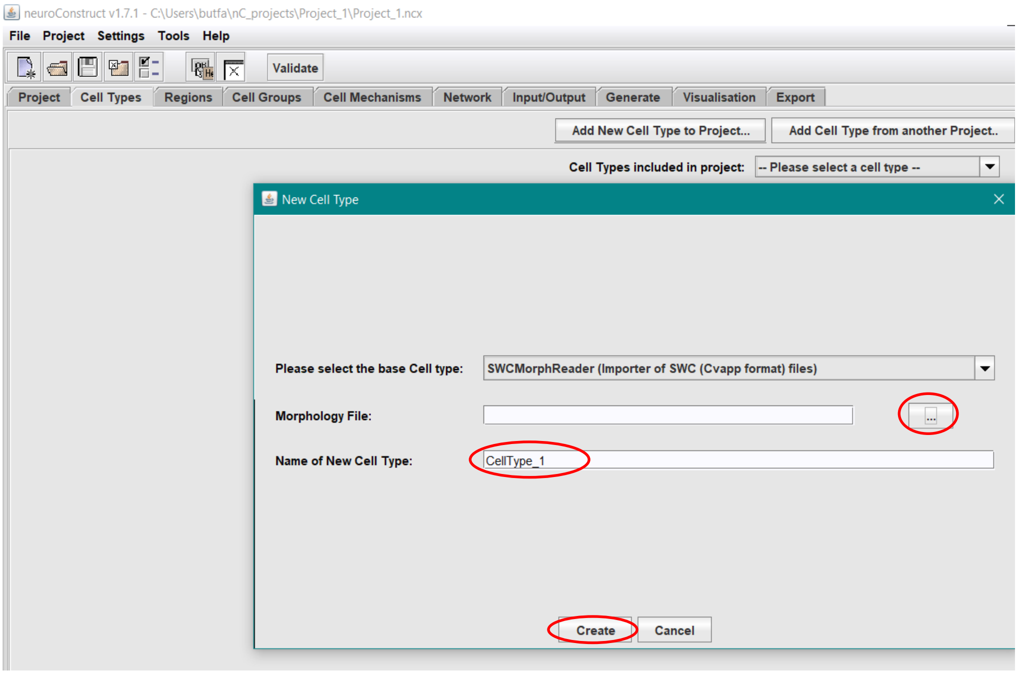

Now, after adding the wanted cell you can add and edit the cell:

Setting cell properties:

Regions:

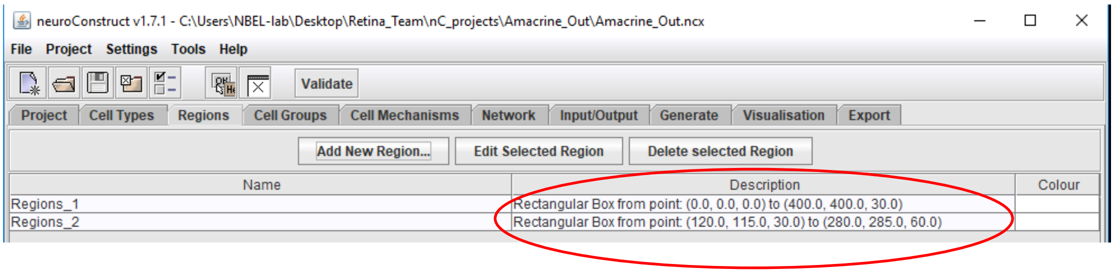

Cell groups:

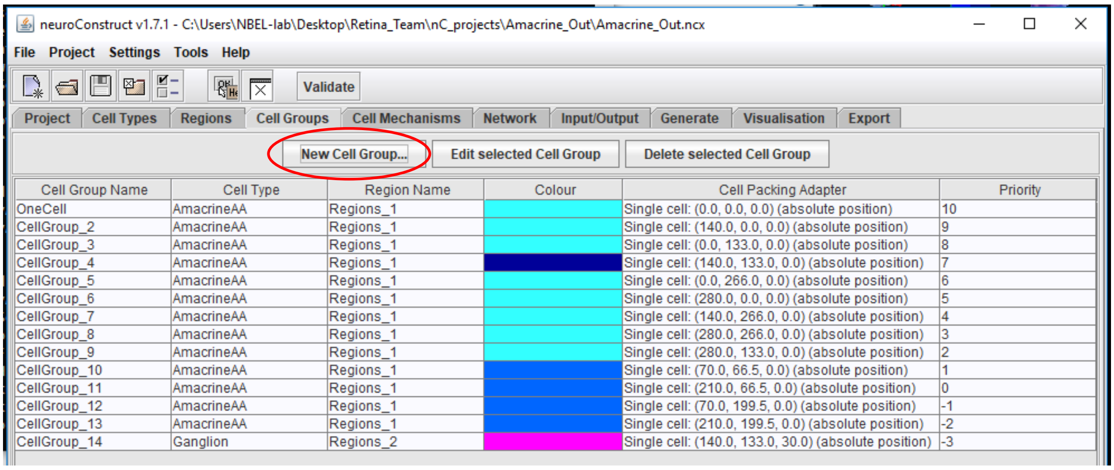

Cell mechanism:

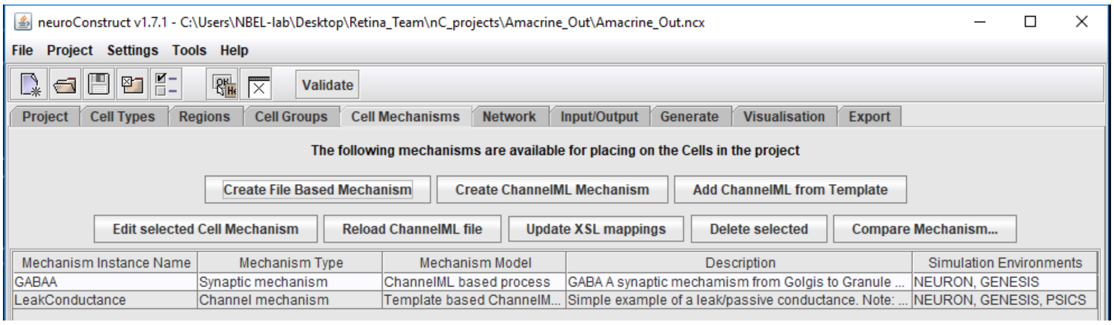

Network:

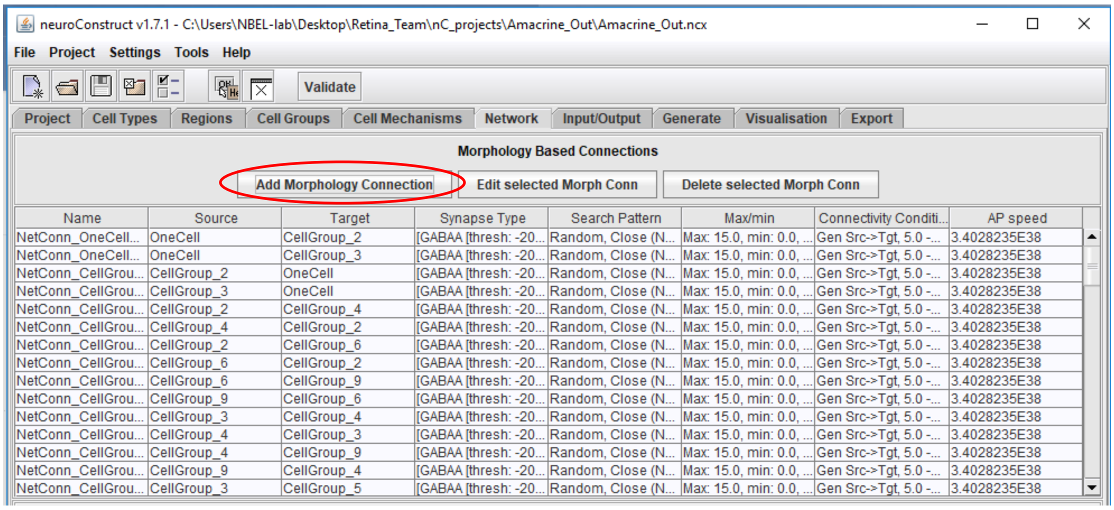

Name	Source	Target	Synapse Type	Search Pattern	Max/min	Connectivity Conditi...	AP speed
NetConn_OneCell...	OneCell	CellGroup_2	[GABAA [thresh: -20...	Random, Close (N...	Max: 15.0, min: 0.0, ...	Gen Src->Tgt, 5.0 -...	3.4028235E38
NetConn_OneCell...	OneCell	CellGroup_3	[GABAA [thresh: -20...	Random, Close (N...	Max: 15.0, min: 0.0, ...	Gen Src->Tgt, 5.0 -...	3.4028235E38
NetConn_CellGrou...	CellGroup_2	OneCell	[GABAA [thresh: -20...	Random, Close (N...	Max: 15.0, min: 0.0, ...	Gen Src->Tgt, 5.0 -...	3.4028235E38
NetConn_CellGrou...	CellGroup_3	OneCell	[GABAA [thresh: -20...	Random, Close (N...	Max: 15.0, min: 0.0, ...	Gen Src->Tgt, 5.0 -...	3.4028235E38
NetConn_CellGrou...	CellGroup_2	CellGroup_4	[GABAA [thresh: -20...	Random, Close (N...	Max: 15.0, min: 0.0, ...	Gen Src->Tgt, 5.0 -...	3.4028235E38
NetConn_CellGrou...	CellGroup_4	CellGroup_2	[GABAA [thresh: -20...	Random, Close (N...	Max: 15.0, min: 0.0, ...	Gen Src->Tgt, 5.0 -...	3.4028235E38
NetConn_CellGrou...	CellGroup_2	CellGroup_6	[GABAA [thresh: -20...	Random, Close (N...	Max: 15.0, min: 0.0, ...	Gen Src->Tgt, 5.0 -...	3.4028235E38
NetConn_CellGrou...	CellGroup_6	CellGroup_2	[GABAA [thresh: -20...	Random, Close (N...	Max: 15.0, min: 0.0, ...	Gen Src->Tgt, 5.0 -...	3.4028235E38
NetConn_CellGrou...	CellGroup_6	CellGroup_9	[GABAA [thresh: -20...	Random, Close (N...	Max: 15.0, min: 0.0, ...	Gen Src->Tgt, 5.0 -...	3.4028235E38
NetConn_CellGrou...	CellGroup_9	CellGroup_6	[GABAA [thresh: -20...	Random, Close (N...	Max: 15.0, min: 0.0, ...	Gen Src->Tgt, 5.0 -...	3.4028235E38
NetConn_CellGrou...	CellGroup_3	CellGroup_4	[GABAA [thresh: -20...	Random, Close (N...	Max: 15.0, min: 0.0, ...	Gen Src->Tgt, 5.0 -...	3.4028235E38
NetConn_CellGrou...	CellGroup_4	CellGroup_3	[GABAA [thresh: -20...	Random, Close (N...	Max: 15.0, min: 0.0, ...	Gen Src->Tgt, 5.0 -...	3.4028235E38
NetConn_CellGrou...	CellGroup_4	CellGroup_9	[GABAA [thresh: -20...	Random, Close (N...	Max: 15.0, min: 0.0, ...	Gen Src->Tgt, 5.0 -...	3.4028235E38
NetConn_CellGrou...	CellGroup_9	CellGroup_4	[GABAA [thresh: -20...	Random, Close (N...	Max: 15.0, min: 0.0, ...	Gen Src->Tgt, 5.0 -...	3.4028235E38
NetConn_CellGrou...	CellGroup_3	CellGroup_5	[GABAA [thresh: -20...	Random, Close (N...	Max: 15.0, min: 0.0, ...	Gen Src->Tgt, 5.0 -...	3.4028235E38

Input/output:

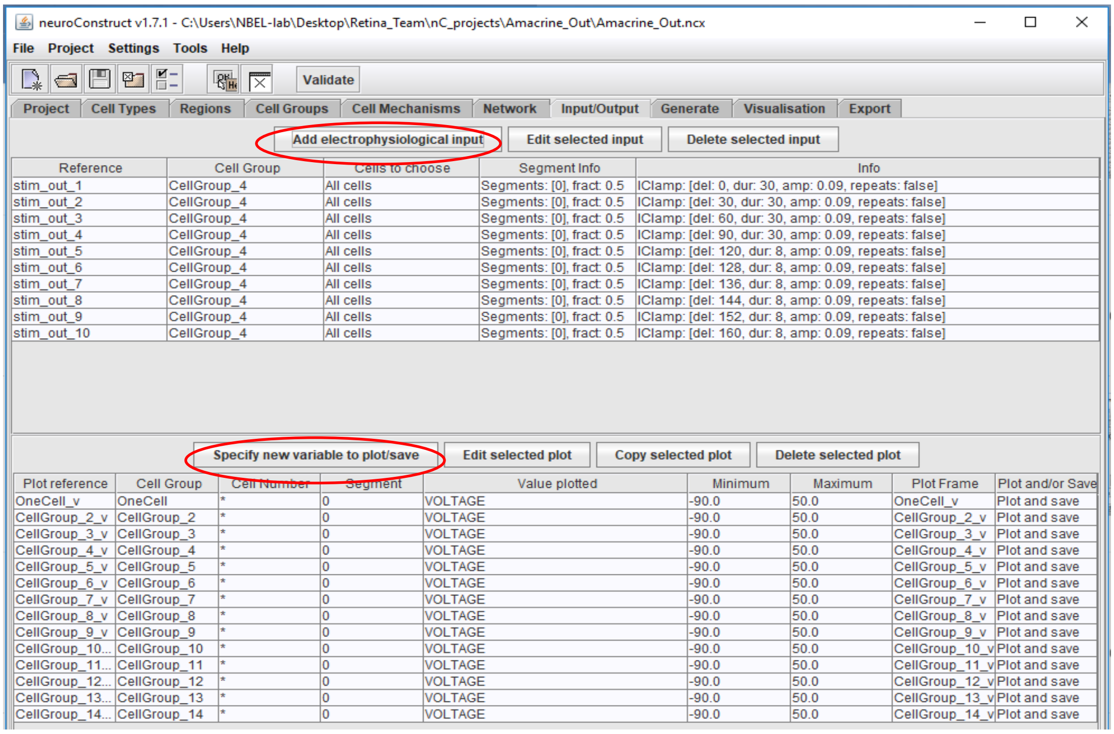

Reference	Cell Group	Cells to choose	Segment Info	Info
stim_out_1	CellGroup_4	All cells	Segments: [0], fract: 0.5	IClamp: [del: 0, dur: 30, amp: 0.09, repeats: false]
stim_out_2	CellGroup_4	All cells	Segments: [0], fract: 0.5	IClamp: [del: 30, dur: 30, amp: 0.09, repeats: false]
stim_out_3	CellGroup_4	All cells	Segments: [0], fract: 0.5	IClamp: [del: 60, dur: 30, amp: 0.09, repeats: false]
stim_out_4	CellGroup_4	All cells	Segments: [0], fract: 0.5	IClamp: [del: 90, dur: 30, amp: 0.09, repeats: false]
stim_out_5	CellGroup_4	All cells	Segments: [0], fract: 0.5	IClamp: [del: 120, dur: 8, amp: 0.09, repeats: false]
stim_out_6	CellGroup_4	All cells	Segments: [0], fract: 0.5	IClamp: [del: 128, dur: 8, amp: 0.09, repeats: false]
stim_out_7	CellGroup_4	All cells	Segments: [0], fract: 0.5	IClamp: [del: 136, dur: 8, amp: 0.09, repeats: false]
stim_out_8	CellGroup_4	All cells	Segments: [0], fract: 0.5	IClamp: [del: 144, dur: 8, amp: 0.09, repeats: false]
stim_out_9	CellGroup_4	All cells	Segments: [0], fract: 0.5	IClamp: [del: 152, dur: 8, amp: 0.09, repeats: false]
stim_out_10	CellGroup_4	All cells	Segments: [0], fract: 0.5	IClamp: [del: 160, dur: 8, amp: 0.09, repeats: false]

Plot reference	Cell Group	Cell Number	Segment	Value plotted	Minimum	Maximum	Plot Frame	Plot and/or Save
OneCell_v	OneCell	*	0	VOLTAGE	-90.0	50.0	OneCell_v	Plot and save
CellGroup_2_v	CellGroup_2	*	0	VOLTAGE	-90.0	50.0	CellGroup_2_v	Plot and save
CellGroup_3_v	CellGroup_3	*	0	VOLTAGE	-90.0	50.0	CellGroup_3_v	Plot and save
CellGroup_4_v	CellGroup_4	*	0	VOLTAGE	-90.0	50.0	CellGroup_4_v	Plot and save
CellGroup_5_v	CellGroup_5	*	0	VOLTAGE	-90.0	50.0	CellGroup_5_v	Plot and save
CellGroup_6_v	CellGroup_6	*	0	VOLTAGE	-90.0	50.0	CellGroup_6_v	Plot and save
CellGroup_7_v	CellGroup_7	*	0	VOLTAGE	-90.0	50.0	CellGroup_7_v	Plot and save
CellGroup_8_v	CellGroup_8	*	0	VOLTAGE	-90.0	50.0	CellGroup_8_v	Plot and save
CellGroup_9_v	CellGroup_9	*	0	VOLTAGE	-90.0	50.0	CellGroup_9_v	Plot and save
CellGroup_10...	CellGroup_10	*	0	VOLTAGE	-90.0	50.0	CellGroup_10_v	Plot and save
CellGroup_11...	CellGroup_11	*	0	VOLTAGE	-90.0	50.0	CellGroup_11_v	Plot and save
CellGroup_12...	CellGroup_12	*	0	VOLTAGE	-90.0	50.0	CellGroup_12_v	Plot and save
CellGroup_13...	CellGroup_13	*	0	VOLTAGE	-90.0	50.0	CellGroup_13_v	Plot and save
CellGroup_14...	CellGroup_14	*	0	VOLTAGE	-90.0	50.0	CellGroup_14_v	Plot and save

Generate:

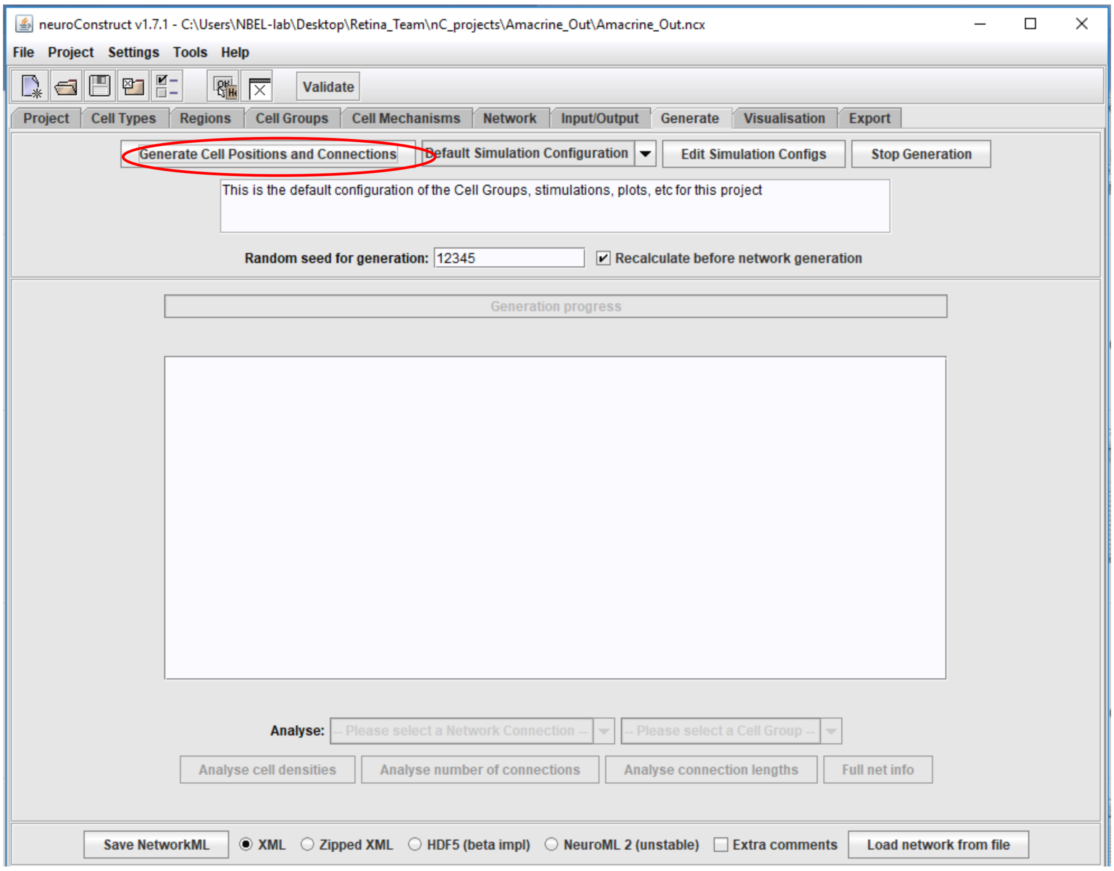

You will see this page

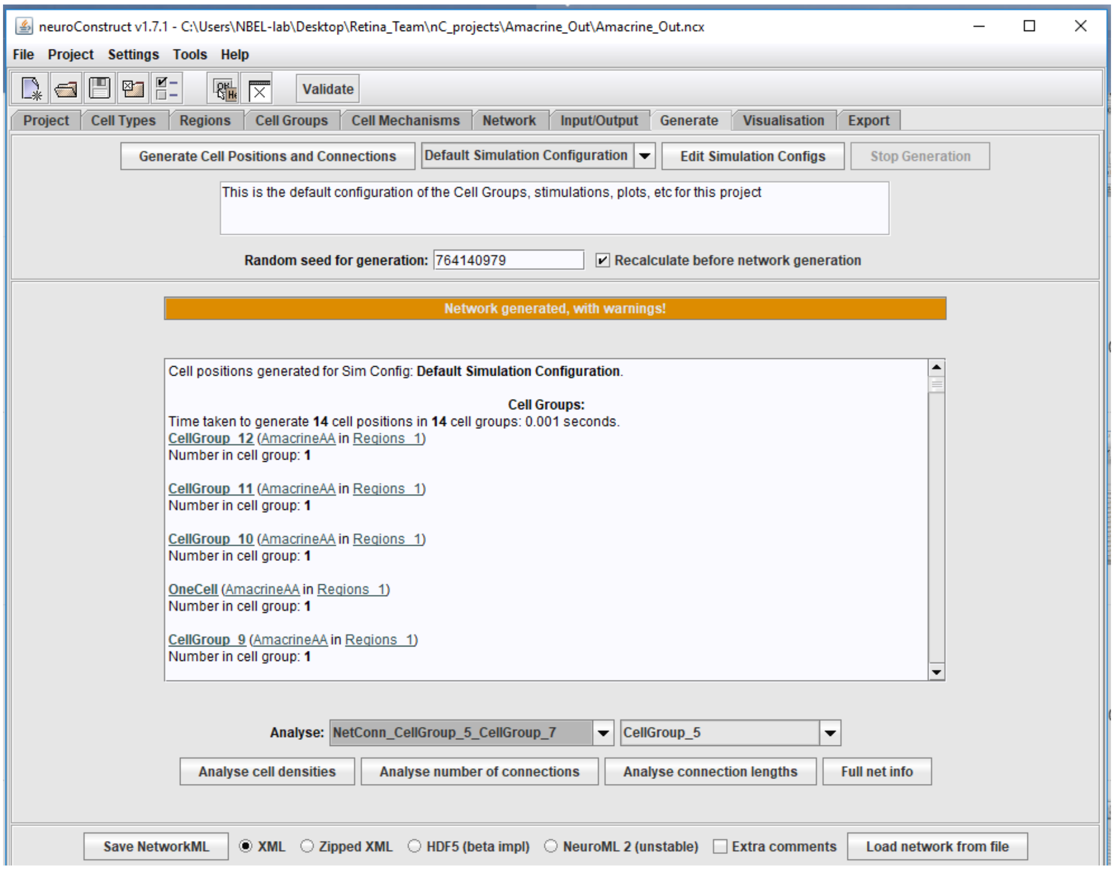

Visualization:

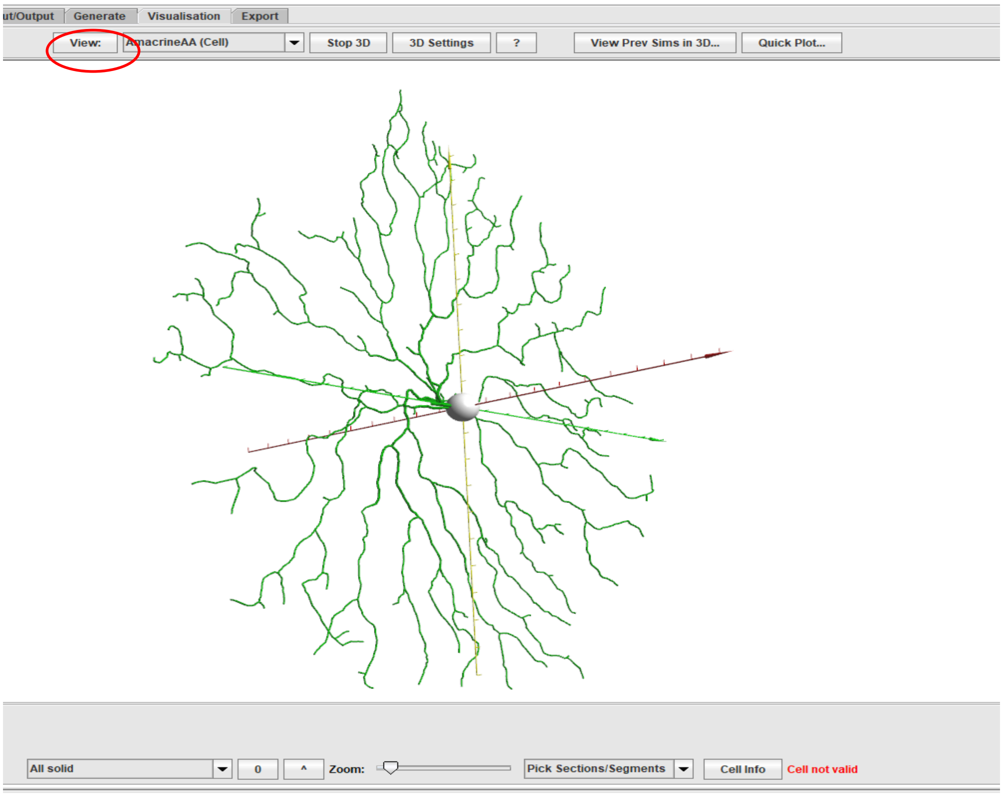

Export*:*

You can set all the properties of your simulation

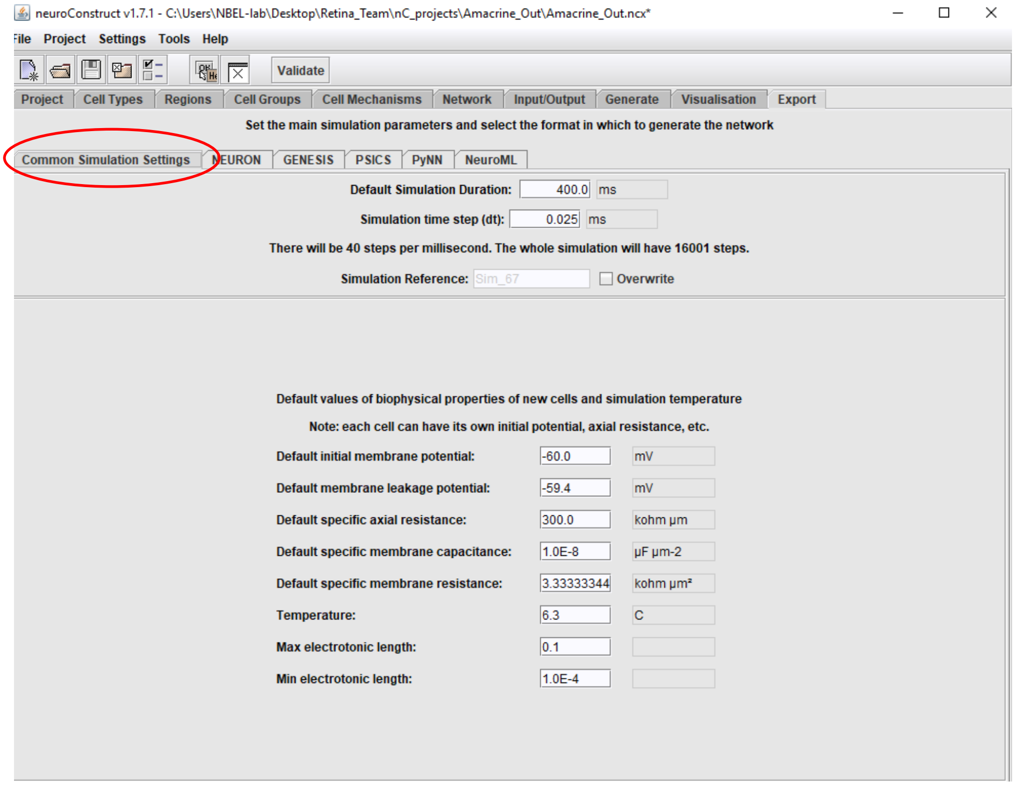

Simulation

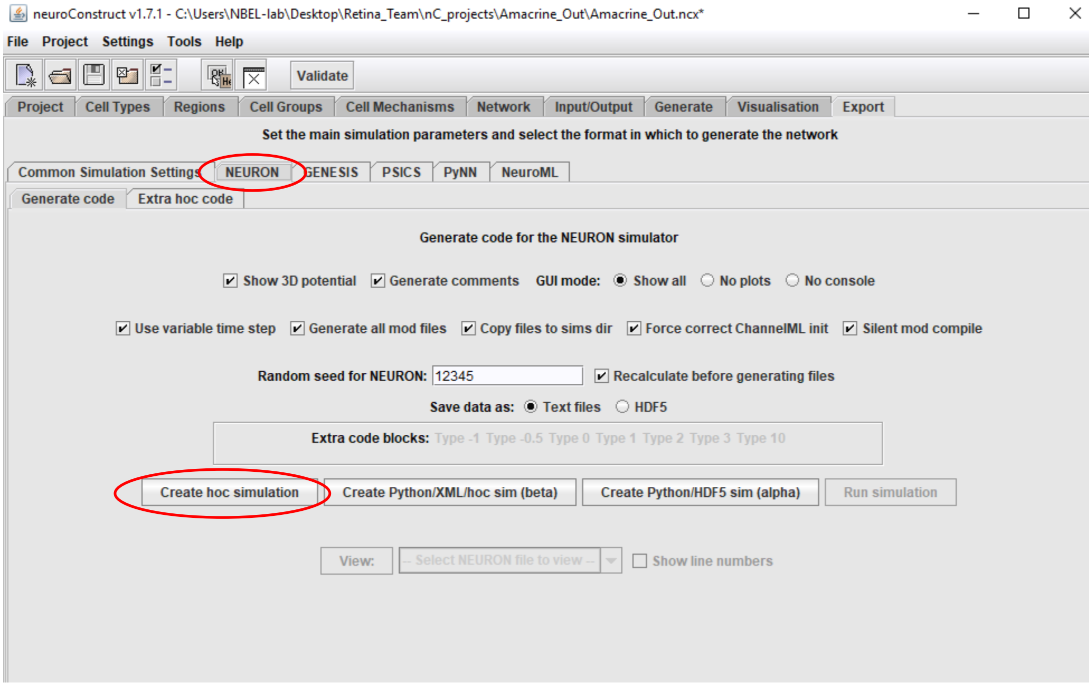
neuroConstruct v1.7.1 - C:\Users\NBEL-lab\Desktop\Retina_Team\nC_projects\Amacrine_Out\Amacrine_Out.ncx*
File Project Settings Tools Help
Validate
Project Cell Types Regions Cell Groups Cell Mechanisms Network Input/Output Generate Visualisation Export
Set the main simulation parameters and select the format in which to generate the network
Common Simulation Settings NEURON GENESIS PSICS PyNN NeuroML
Generate code Extra hoc code
Generate code for the NEURON simulator
Show 3D potential Generate comments GUI mode: Show all No plots No console
Use variable time step Generate all mod files Copy files to sims dir Force correct ChannelML init Silent mod compile
Random seed for NEURON: 12345 Recalculate before generating files
Save data as: Text files HDF5
Extra code blocks: Type -1 Type -0.5 Type 0 Type 1 Type 2 Type 3 Type 10
Create hoc simulation
Create Python/XML/hoc sim (beta)
Create Python/HDF5 sim (alpha)
Run simulation
View: -- Select NEURON file to view -- Show line numbers

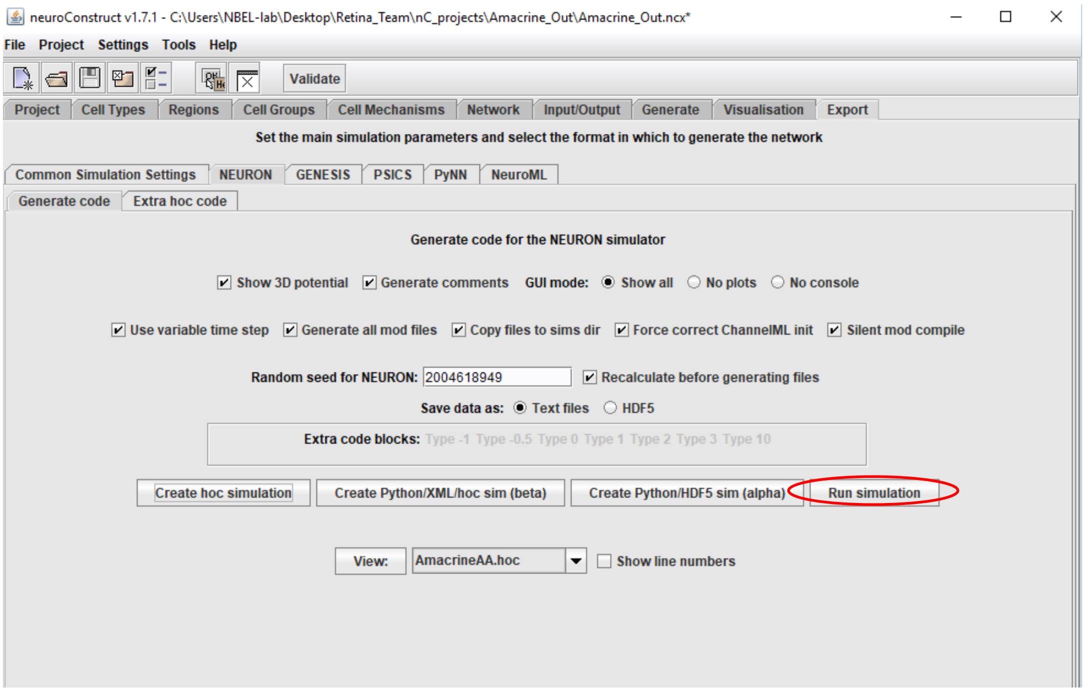
neuroConstruct v1.7.1 - C:\Users\NBEL-lab\Desktop\Retina_Team\nC_projects\Amacrine_Out\Amacrine_Out.ncx*
File Project Settings Tools Help
Validate
Project Cell Types Regions Cell Groups Cell Mechanisms Network Input/Output Generate Visualisation Export
Set the main simulation parameters and select the format in which to generate the network
Common Simulation Settings NEURON GENESIS PSICS PyNN NeuroML
Generate code Extra hoc code
Generate code for the NEURON simulator
Show 3D potential Generate comments GUI mode: Show all No plots No console
Use variable time step Generate all mod files Copy files to sims dir Force correct ChannelML init Silent mod compile
Random seed for NEURON: 2004618949 Recalculate before generating files
Save data as: Text files HDF5
Extra code blocks: Type -1 Type -0.5 Type 0 Type 1 Type 2 Type 3 Type 10
Create hoc simulation
Create Python/XML/hoc sim (beta)
Create Python/HDF5 sim (alpha)
Run simulation
View: AmacrineAA.hoc Show line numbers

<u>Following simulation</u> in NEURON you can the following results

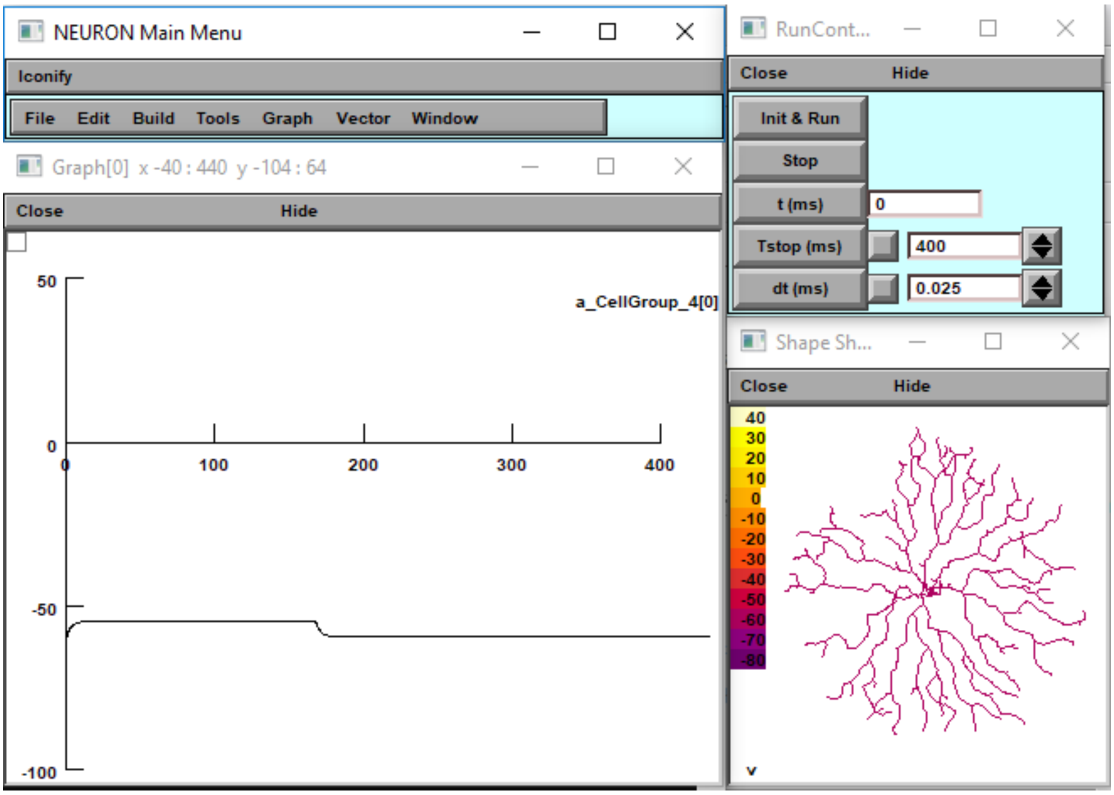

Extending neuroConstruct

SAC morphology is exported as nml file:

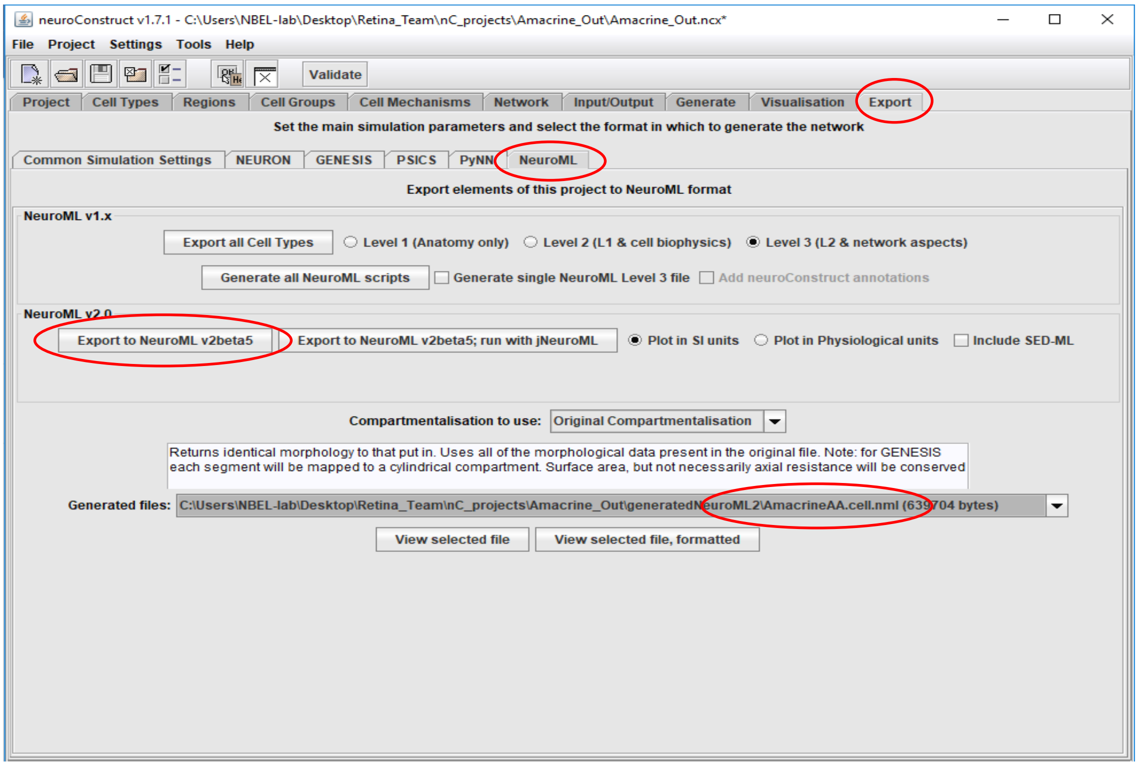

Generate a NeuroML script:

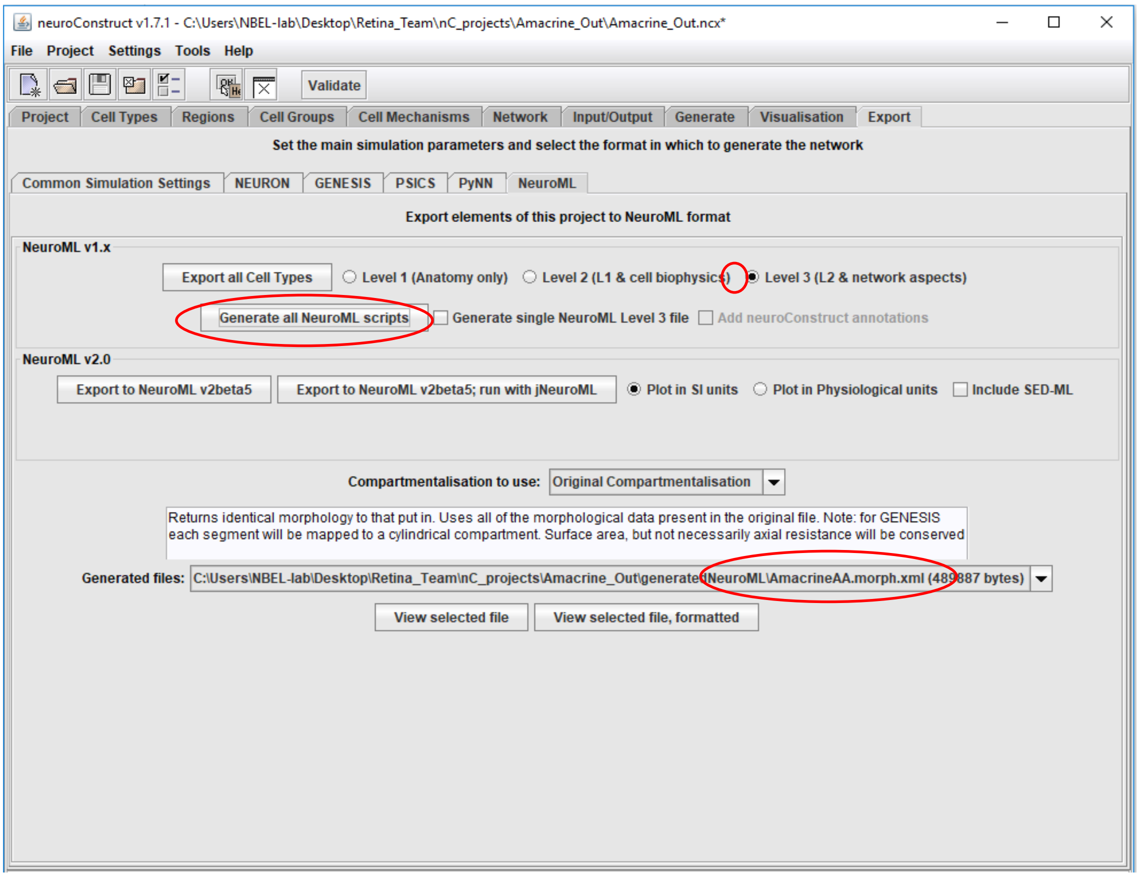

The following are code examples which introduce key aspects in our framework

The following python script divides a given cell into several sectional, groups based on section's distance from the soma. The script takes the nml file and creates 3 groups of sections, and then takes the file xml and export it to a new one which includes the new groups.

```
#define 3 groups in order to devide the cell into 3 groups
#based on the distance from the cell soma
    cellWidth=input('Enter the cell width: ')
    dist=cellWidth/4;
    g1=[]
    g2=[]
    g3=[]
    for key in special:
        if seg_len_d[key]<dist:
            g1.append(key)
        elif seg_len_d[key]<2*dist:
            g2.append(key)
        elif seg_len_d[key]<3*dist:
            g3.append(key)
```

The following creates a new XML file:

```
c1=[]
c2=[]
c3=[]

for elem in tree.iter(tag='{http://morphml.org/morphml/schema}segment'):
    if (int(elem.attrib['id']) in g1) and not(elem.attrib['cable'] in c1):
        c1.append(elem.attrib['cable'])
    elif (int(elem.attrib['id']) in g2) and not(elem.attrib['cable'] in c2):
        c2.append(elem.attrib['cable'])
    elif (int(elem.attrib['id']) in g3) and not(elem.attrib['cable'] in c3):
        c3.append(elem.attrib['cable'])

for elem in tree.iter(tag='{http://morphml.org/morphml/schema}cable'):
    child = xml.SubElement(elem, "{http://morphml.org/metadata/schema}group")
    if(elem.attrib['id'] in c1):
        child.text='G_1'
    elif(elem.attrib['id'] in c2):
        child.text='G_2'
    elif(elem.attrib['id'] in c3):
        child.text='G_3'
```

The following is an example of the generated XML:

```
<ns3:cable id="2" name="Sec_Comp_10">
    <ns2:group>all</ns2:group>
    <ns2:group>dendrite_group</ns2:group>
    <ns2:group>Colour_Green</ns2:group>
<ns2:group>G_1</ns2:group></ns3:cable>

<ns3:cable id="3" name="Sec_Comp_18">
    <ns2:group>all</ns2:group>
    <ns2:group>dendrite_group</ns2:group>
    <ns2:group>Colour_Green</ns2:group>
<ns2:group>G_2</ns2:group></ns3:cable>

<ns3:cable id="4" name="Sec_Comp_31">
    <ns2:group>all</ns2:group>
    <ns2:group>dendrite_group</ns2:group>
    <ns2:group>Colour_Green</ns2:group>
<ns2:group>G_3</ns2:group></ns3:cable>
```

In neuroConstruct, we load the script by the following:
cellTypes -> Add new cellTypes->NeuroMLConverter->morphology file: file_name->name: AmacrineAA (AmacrineA is the amacrine cell which is examined now) ->create.

We created an amacrine cell, which has its segments divided into 3 groups.

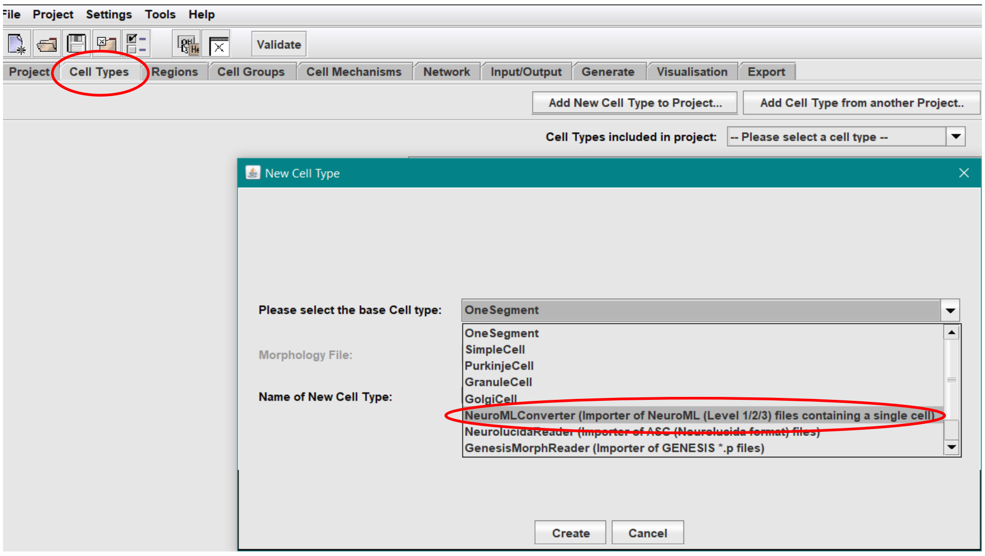

Choose in cell type NeuroML converter:

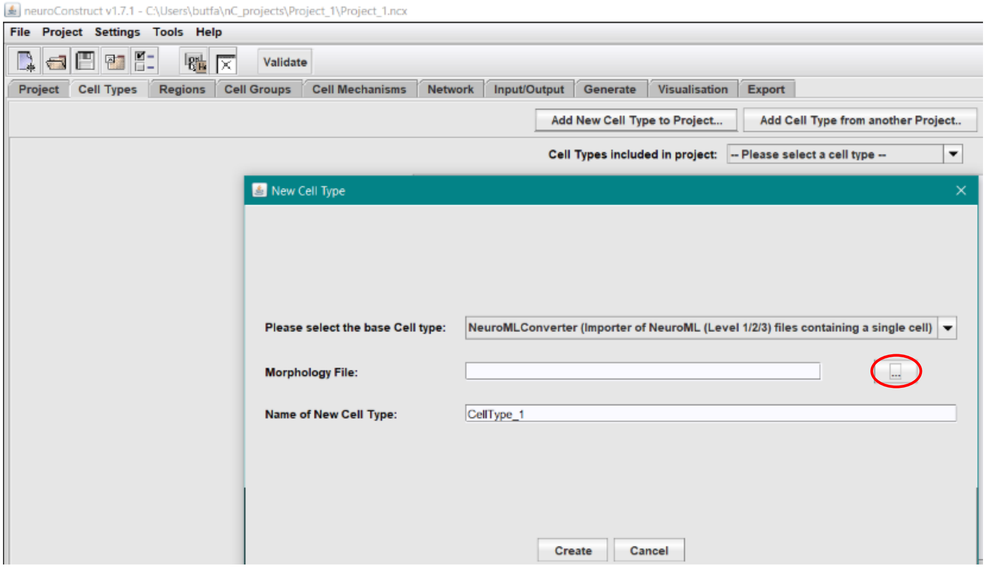

Visualization

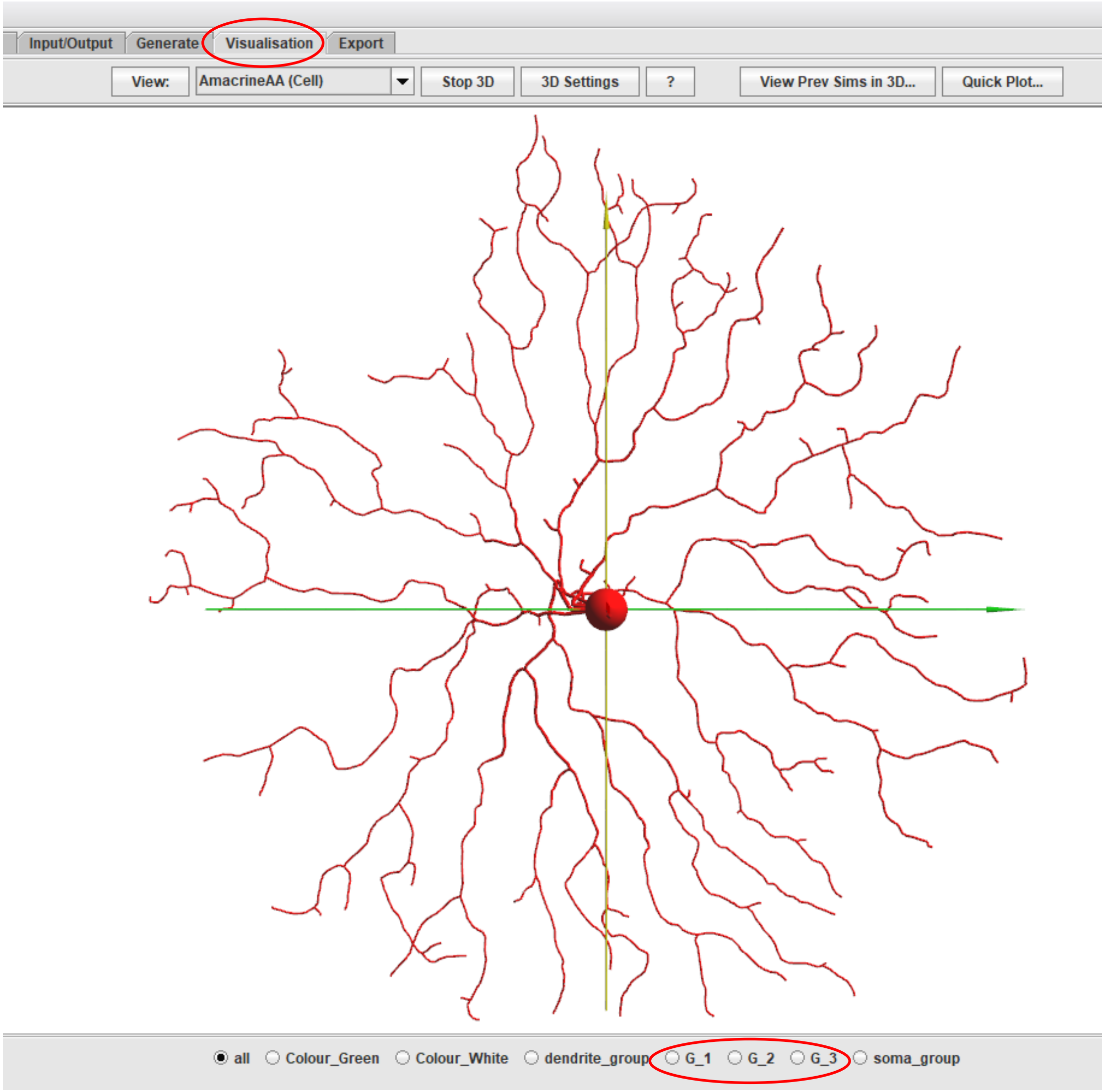

Each group can now be examined separately:

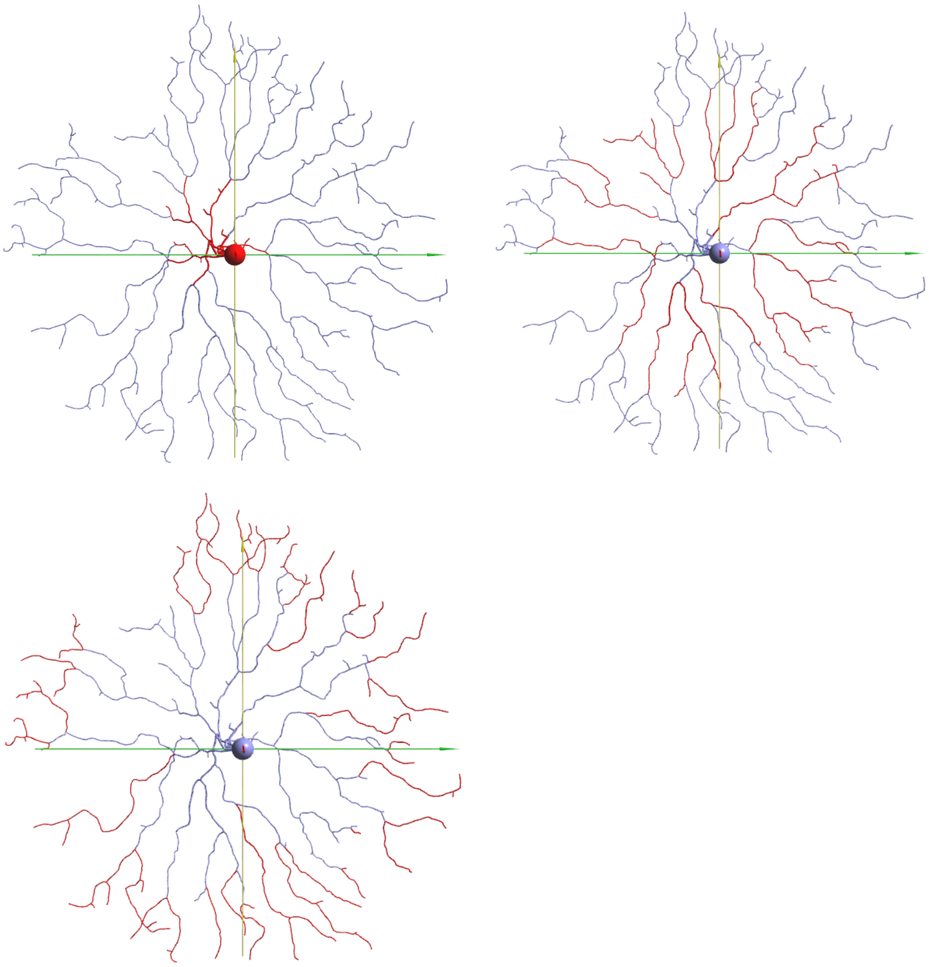

Now we can set different properties for each group. For example, stimuli with different currents

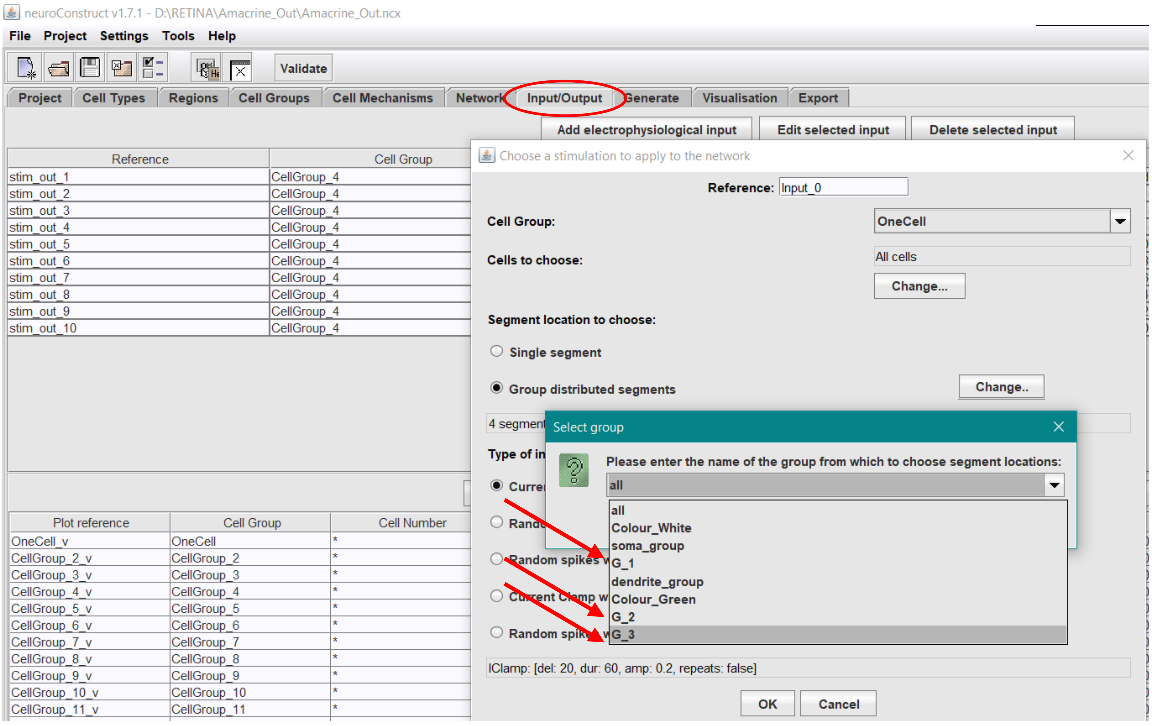

We added different current on the three parts of the cell

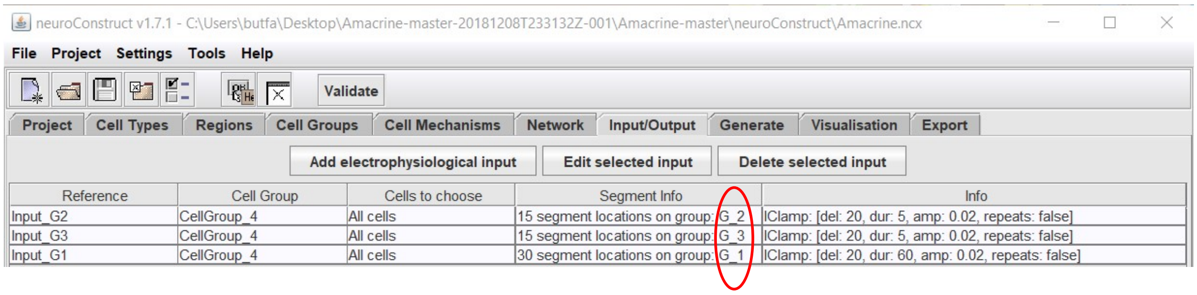

The input for the amacrine cell is composed of 2 types one is for the two thirds of the external cell, and the second is for the inner third.

We can carry on with the creation a 3 x 3 SACs network.

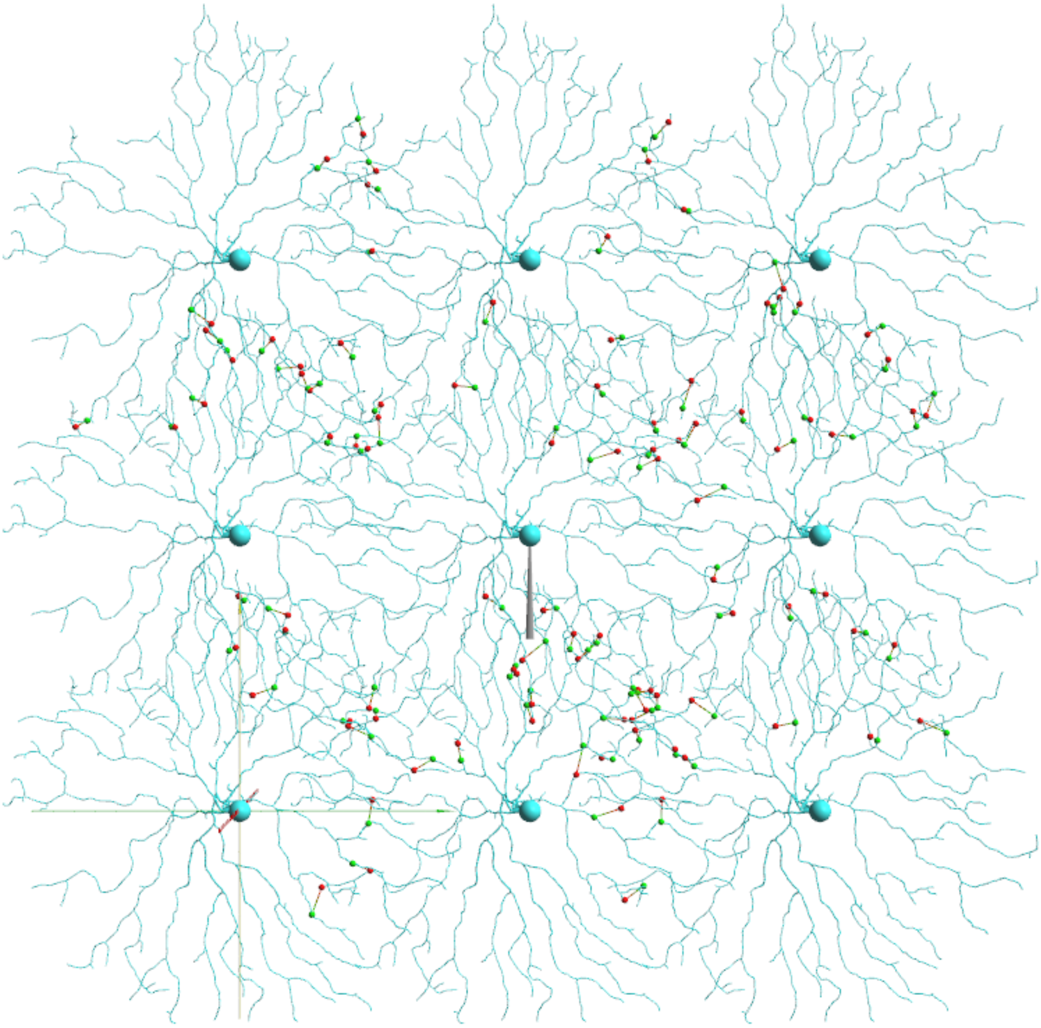

Stimulating the network:

To stimulate the middle amacrine cell we export it from the generate tab:

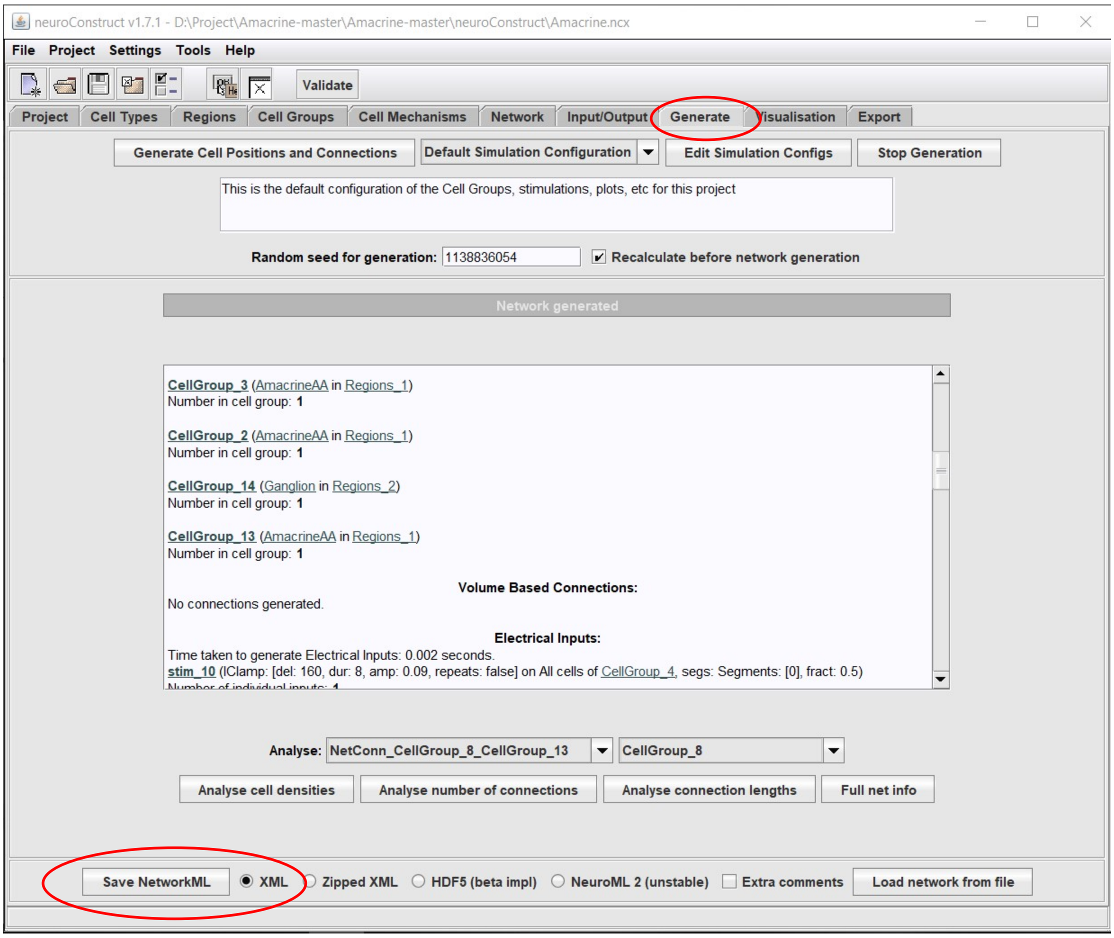

The following script will generate a moving visual stimulus, generated across 10 groups of cell segments. The following visualizes the locations for current injections:

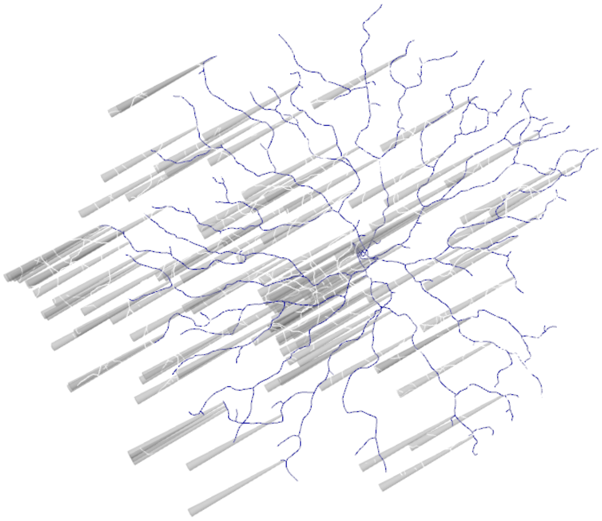

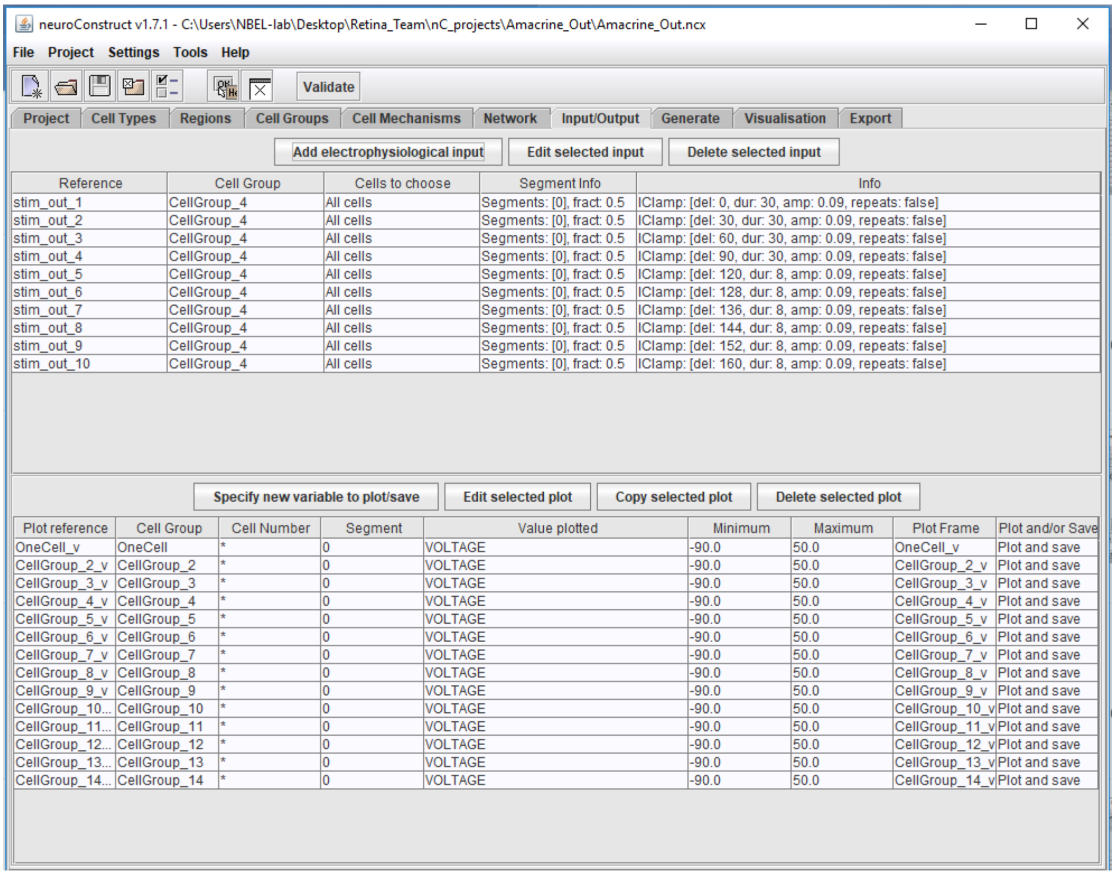

Reference	Cell Group	Cells to choose	Segment Info	Info
stim_out_1	CellGroup_4	All cells	Segments: [0], fract: 0.5	IClamp: [del: 0, dur: 30, amp: 0.09, repeats: false]
stim_out_2	CellGroup_4	All cells	Segments: [0], fract: 0.5	IClamp: [del: 30, dur: 30, amp: 0.09, repeats: false]
stim_out_3	CellGroup_4	All cells	Segments: [0], fract: 0.5	IClamp: [del: 60, dur: 30, amp: 0.09, repeats: false]
stim_out_4	CellGroup_4	All cells	Segments: [0], fract: 0.5	IClamp: [del: 90, dur: 30, amp: 0.09, repeats: false]
stim_out_5	CellGroup_4	All cells	Segments: [0], fract: 0.5	IClamp: [del: 120, dur: 8, amp: 0.09, repeats: false]
stim_out_6	CellGroup_4	All cells	Segments: [0], fract: 0.5	IClamp: [del: 128, dur: 8, amp: 0.09, repeats: false]
stim_out_7	CellGroup_4	All cells	Segments: [0], fract: 0.5	IClamp: [del: 136, dur: 8, amp: 0.09, repeats: false]
stim_out_8	CellGroup_4	All cells	Segments: [0], fract: 0.5	IClamp: [del: 144, dur: 8, amp: 0.09, repeats: false]
stim_out_9	CellGroup_4	All cells	Segments: [0], fract: 0.5	IClamp: [del: 152, dur: 8, amp: 0.09, repeats: false]
stim_out_10	CellGroup_4	All cells	Segments: [0], fract: 0.5	IClamp: [del: 160, dur: 8, amp: 0.09, repeats: false]

Plot reference	Cell Group	Cell Number	Segment	Value plotted	Minimum	Maximum	Plot Frame	Plot and/or Save
OneCell_v	OneCell	*	0	VOLTAGE	-90.0	50.0	OneCell_v	Plot and save
CellGroup_2_v	CellGroup_2	*	0	VOLTAGE	-90.0	50.0	CellGroup_2_v	Plot and save
CellGroup_3_v	CellGroup_3	*	0	VOLTAGE	-90.0	50.0	CellGroup_3_v	Plot and save
CellGroup_4_v	CellGroup_4	*	0	VOLTAGE	-90.0	50.0	CellGroup_4_v	Plot and save
CellGroup_5_v	CellGroup_5	*	0	VOLTAGE	-90.0	50.0	CellGroup_5_v	Plot and save
CellGroup_6_v	CellGroup_6	*	0	VOLTAGE	-90.0	50.0	CellGroup_6_v	Plot and save
CellGroup_7_v	CellGroup_7	*	0	VOLTAGE	-90.0	50.0	CellGroup_7_v	Plot and save
CellGroup_8_v	CellGroup_8	*	0	VOLTAGE	-90.0	50.0	CellGroup_8_v	Plot and save
CellGroup_9_v	CellGroup_9	*	0	VOLTAGE	-90.0	50.0	CellGroup_9_v	Plot and save
CellGroup_10...	CellGroup_10	*	0	VOLTAGE	-90.0	50.0	CellGroup_10_v	Plot and save
CellGroup_11...	CellGroup_11	*	0	VOLTAGE	-90.0	50.0	CellGroup_11_v	Plot and save
CellGroup_12...	CellGroup_12	*	0	VOLTAGE	-90.0	50.0	CellGroup_12_v	Plot and save
CellGroup_13...	CellGroup_13	*	0	VOLTAGE	-90.0	50.0	CellGroup_13_v	Plot and save
CellGroup_14...	CellGroup_14	*	0	VOLTAGE	-90.0	50.0	CellGroup_14_v	Plot and save

The following script examples generate the collapsing and extending rings of light

Collapsing

```
root = ET.Element("inputs");
root.tail="\n";
#for case 1
stims=[1,2,3,4,5,6,7,8,9,10];
delayes=[0,30,60,90,120,128,136,144,152,160];
dur=[30,30,30,30,8,8,8,8,8,8];

for i in range(10):
    inputs= ET.SubElement(root,"input", name="stim_out_%s"%(stims[i]));
    inputs.tail="\n";
    pulse= ET.SubElement(inputs, "pulse_input", delay="%s"%delayes[i], duration="%s"%dur[i], amplitude="9.0E-5" );
    pulse.tail="\n";
    target = ET.SubElement(inputs, "target",population="CellGroup_4" );
    target.tail="\n";
    sites=ET.SubElement(target, "sites", size="10");
    sites.tail="\n";
    for seg_id in segArray_list[(9-i)]:
        ss=ET.SubElement(sites, "site", cell_id="0", segment_id="%s"%seg_id, fraction_along="0.5");
        ss.tail="\n";


tree = ET.ElementTree(root);
tree.write("Stim_Out.xml");
```

Expanding

```
root = ET.Element("inputs");
root.tail="\n";
#for case 1
stims=[1,2,3,4,5,6,7,8,9,10];
delayes=[0,30,60,90,120,128,136,144,152,160];
dur=[30,30,30,30,8,8,8,8,8,8];

for i in range(10):
    inputs= ET.SubElement(root,"input", name="stim_%s"%stims[i]);
    inputs.tail="\n";
    pulse= ET.SubElement(inputs, "pulse_input", delay="%s"%delayes[i], duration="%s"%dur[i], amplitude="9.0E-5" );
    pulse.tail="\n";
    target = ET.SubElement(inputs, "target",population="CellGroup_4" );
    target.tail="\n";
    sites=ET.SubElement(target, "sites", size="10");
    sites.tail="\n";
    for seg_id in segArray_list[i]:
        ss=ET.SubElement(sites, "site", cell_id="0", segment_id="%s"%seg_id, fraction_along="0.5");
        ss.tail="\n";



tree = ET.ElementTree(root);
tree.write("Stim_In.xml");
```

From this scripts, new nml files are generated as was previously described.

Generating SACs plexus

We added another layer of 2x2 SACs as was previously describes.

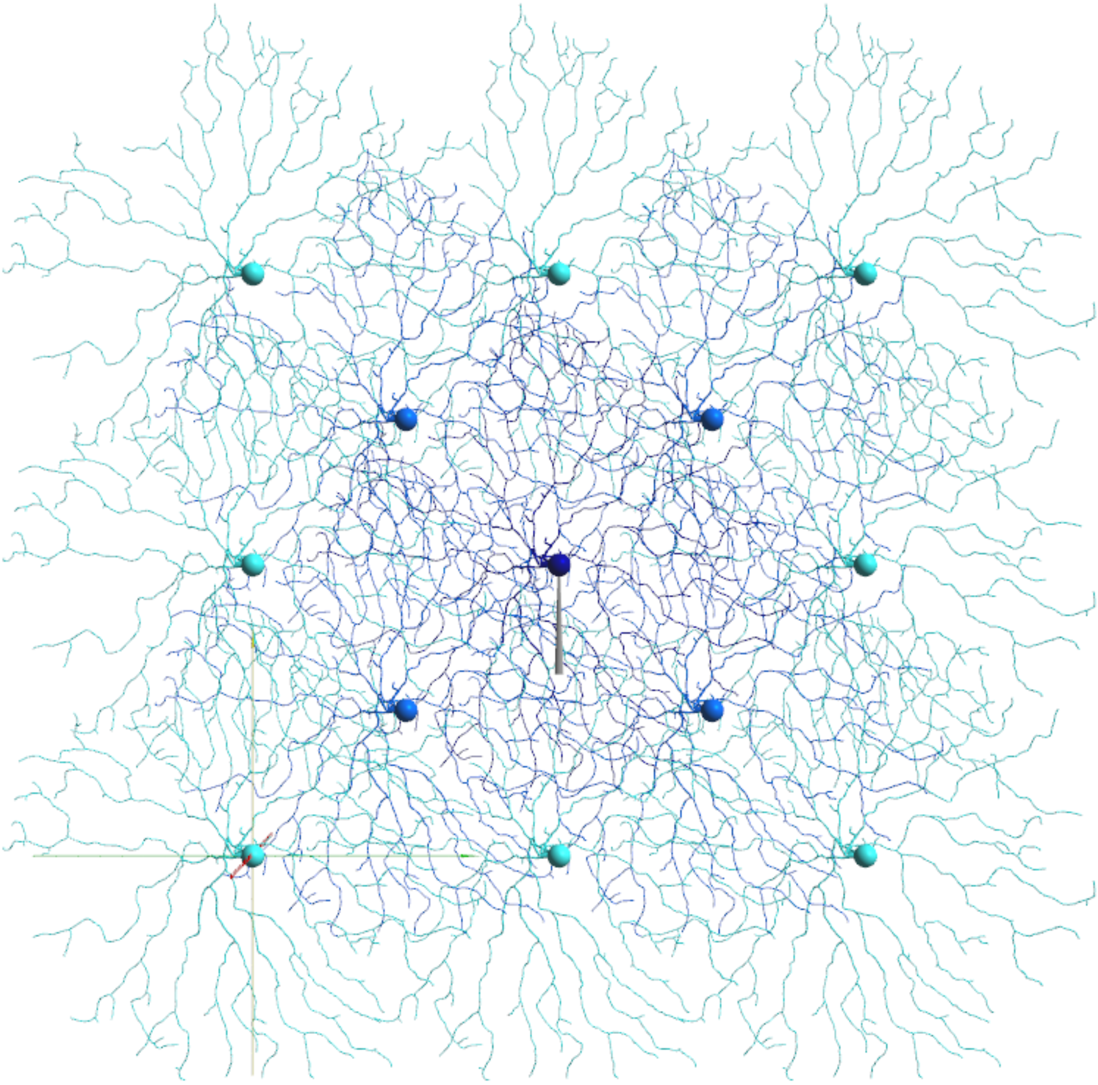

Adding a ganglion cell to the network

We added a ganglion cell to the simulation, as was previously described for the SAC. The ganglion cell morphology was adopted from: http://neuromorpho.org/neuron_info.jsp?neuron_name=T40-2

The a ganglion cell was vertically aligned against the SAC plexus. Synapses were formed as it is described in the text, according to cosine similarity.

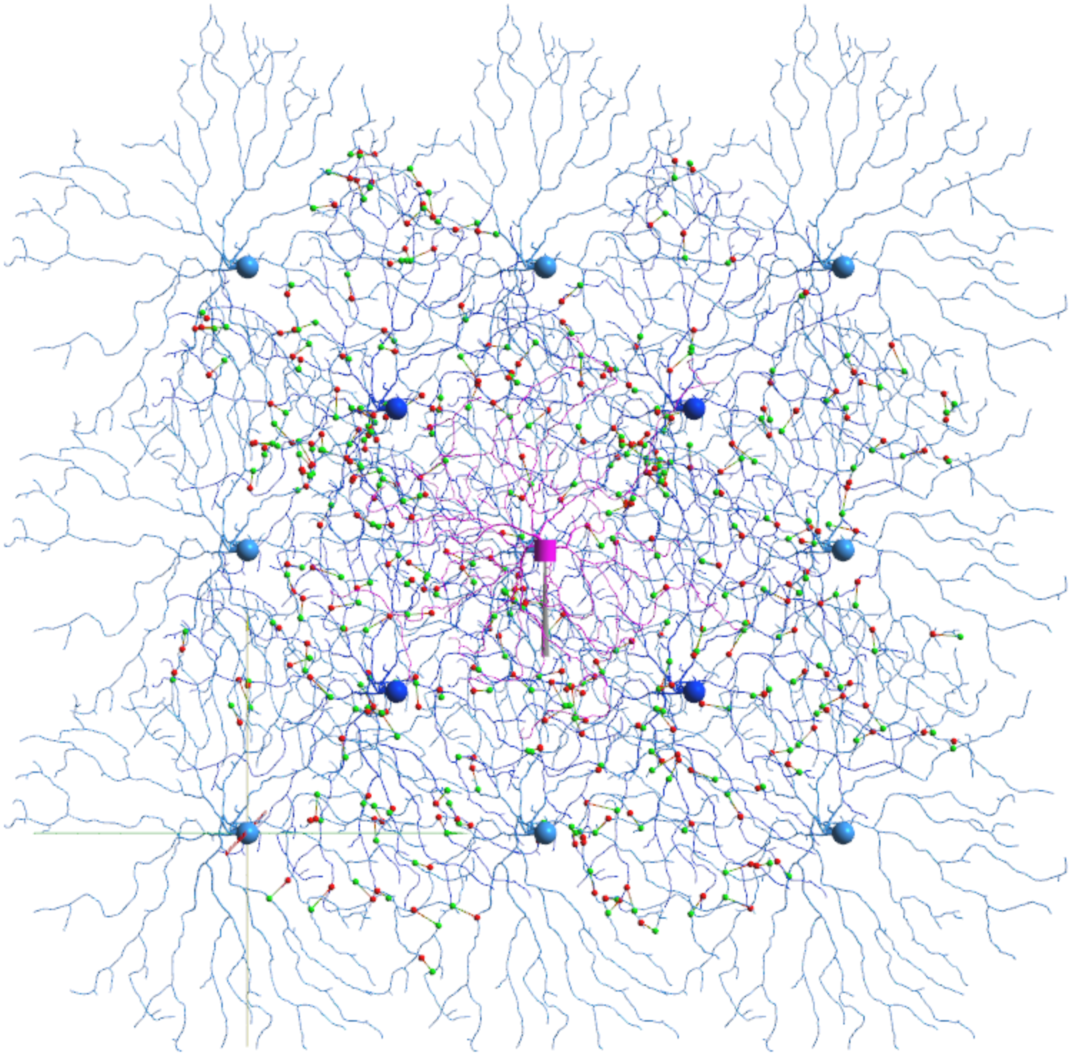

Connections between the SACs and the Ganglion cell are defined using the cosine similarity measure (see text for details). The following defines the synapses according to the cosine similarity:

```
# -*- coding: utf-8 -*-
"""
Created on Mon Mar 11 15:13:04 2019

@author: NBEL-lab
"""

import random
import numpy as np
from sklearn.metrics.pairwise import cosine_similarity
import coord_R


# A function that returns the segments in which we create synapses
#according to the cosine similarity of the vectors
def distance(cell_1fn,c1,cell_2fn,c2,dMin):
 cell_1=coord_R.coord(cell_1fn,c1)
 cell_2=coord_R.coord(cell_2fn,c2)
 syn=[]
 for idA,a1 in cell_1.items():
     for idB,b1 in cell_2.items():
         a=np.asarray(a1)
         b=np.asarray(b1)
         #cosine similarity
         dot = np.dot(a, b)
         norma = np.linalg.norm(a)
         normb = np.linalg.norm(b)
         cos = dot / (norma * normb)
         #distance between vectors
         dis=np.linalg.norm(a-b)
         #probability
         #sim=1/cos
         ran=float(random.random())
         if dis<dMin and ran<abs(cos):
             syn.append([idA,idB])
 return(syn)
```
