# Supplementary material for: NeuroConstruct-based implementation of structured-light stimulated retinal circuitry
Source: BMC Neurosci. 2020 Jun 24;21:28. doi: 10.1186/s12868-020-00578-0 (PMC7315481; doi:10.1186/s12868-020-00578-0)
Supplement: Supplementary file 2 — Additional file 2. Code example 1: Network creation. [file 12868_2020_578_MOESM2_ESM.pdf]

```python
import neuroml
import math
import neuroml.loaders as loaders
import neuroml.writers as writers
from Functions import analyse
from pyneuroml.lems import generate_lems_file_for_neuroml

net_ref = "Amacrine_StimNet"
net_doc = neuroml.NeuroMLDocument(id=net_ref)

net = neuroml.Network(id=net_ref)
net_doc.networks.append(net)

cell_name = 'AmacrineAA'
cell_id = cell_name

net_doc.includes.append(neuroml.IncludeType(cell_id+'.cell.nml'))

pop = neuroml.Population(id="popA",
            component=cell_id,
            type="populationList")

inst = neuroml.Instance(id="0")
pop.instances.append(inst)
inst.location = neuroml.Location(x=0, y=0, z=0)
net.populations.append(pop)

if __name__ == '__main__':
    fn = 'AmacrineAAcoord_pro = [g.x, g.y].cell.nml'
    g1,g2,g3,g4,g5,g6,g7,g8,g9,g10 = analyse(fn)

stim_delays_seg_id = {'50ms':0, '200ms':500, '350ms':1000, '500ms':1500, '650ms':2000}

for delay in stim_delays_seg_id:

    seg_id = stim_delays_seg_id[delay]

    for t in range(100):
        if t<=15:
            stim0 = neuroml.PulseGenerator(id='stim_%s'%delay,
```

```
                                    delay='0ms',
                                    duration='15ms',
                                    amplitude='%f nA'%(math.exp(0.35322*t)))
        elif t<=60:
            stim0 = neuroml.PulseGenerator(id='stim_%s'%delay,
                                    delay='15ms',
                                    duration='45ms',
                                    amplitude='%f nA'%(100+math.exp(-0.7*t+15)))
        elif t<70:
            stim0 = neuroml.PulseGenerator(id='stim_%s'%delay,
                                    delay='60ms',
                                    duration='10ms',
                                    amplitude='%f nA'%(100+1/(t-70.01)))
        elif t<=100:
            stim0 = neuroml.PulseGenerator(id='stim_%s'%delay,
                                    delay='70ms',
                                    duration='30ms',
                                    amplitude='%f nA'%(10+math.exp(-t+73)))
        net_doc.pulse_generators.append(stim0)


    input_list0 = neuroml.InputList(id="%s_input"%stim0.id,
                                    component=stim0.id,
                                    populations=pop.id)
    net.input_lists.append(input_list0)

    syn_input0 = neuroml.Input(id=0,
                               target="../%s/0/%s" % (pop.id, pop.component),
                               segment_id=seg_id,
                               destination="synapses")

    input_list0.input.append(syn_input0)

nml_file = net.id+'.net.nml'

writers.NeuroMLWriter.write(net_doc,nml_file)

print("Saved network file to: "+nml_file)
```

```python
###### Validate  NeuroML ######

from neuroml.utils import validate_neuroml2

validate_neuroml2(nml_file)

sim_id = 'TestInputs'
target = net.id
duration=800
dt = 0.025
lems_file_name = 'LEMS_%s.xml'%sim_id
target_dir = "."

interesting_seg_ids = [0,500,1000,1500,2000]

to_plot = {'Some_voltages':[]}
to_save = {'%s_voltages.dat'%cell_id:[]}

to_p = list(to_plot.values())
to_s = list(to_save.values())

for seg_id in interesting_seg_ids:
    to_p[0].append('%s/0/%s/%s/v'%(pop.id, pop.component,seg_id))
    to_s[0].append('%s/0/%s/%s/v'%(pop.id, pop.component,seg_id))

generate_lems_file_for_neuroml(sim_id,
                               nml_file,
                               target,
                               duration,
                               dt,
                               lems_file_name,
                               target_dir,
                               gen_plots_for_all_v = False,
                               plot_all_segments = False,
                               gen_plots_for_quantities = to_plot,
                               gen_saves_for_all_v = False,
                               save_all_segments = False,
                               gen_saves_for_quantities = to_save,
                               copy_neuroml = False)
```
