# Supplementary material for: NeuroConstruct-based implementation of structured-light stimulated retinal circuitry
Source: BMC Neurosci. 2020 Jun 24;21:28. doi: 10.1186/s12868-020-00578-0 (PMC7315481; doi:10.1186/s12868-020-00578-0)
Supplement: Supplementary file 3 — Additional file 3. Code example 2: Synapse creation and distribution. [file 12868_2020_578_MOESM3_ESM.pdf]

```python
"""
Created on Wed Mar 13 10:52:57 2019
"""

from Functions import distance
import random
import xml.etree.cElementTree as ET
import xml.etree.ElementTree as xml

# Code that creates the synapses XML file
if __name__ == '__main__':
    cell_1fn = 'AmacrineAA.cell.nml'
    cell_2fn='GanglionGG.cell.nml'
    cA={"OneCell":[0,0,0],
        "CellGroup_2":[130,0,0],"CellGroup_3":[0,125,0],"CellGroup_4":[130,125,0],
        "CellGroup_5":[0,250,0],"CellGroup_6":[260,0,0],"CellGroup_7":[130,250,0],
        "CellGroup_8":[260,250,0],"CellGroup_9":[260,125,0],"CellGroup_10":[65,62.5,0],
        "CellGroup_11":[195,62.5,0],"CellGroup_12":[65,187.5,0],"CellGroup_13":
[195,187.5,0]}

    cG=[130,125,15]
    Dmin=4
    syns={}
    for ka,va in cA.items():
        syns[ka] = distance(cell_1fn,va,cell_2fn,cG,Dmin)

    # xml
    root = ET.Element("projections")
    root.tail="\n"

    for ks,vs in syns.items():
        if len(vs)>0:
            pro= ET.SubElement(root,"projection",
                               name="NetConn_%s_CellGroup_14"%ks,
                               source="%s"%ks,
                               target="CellGroup_14")
            pro.tail="\n"
            syn_pro= ET.SubElement(pro, "synapse_props",
                                   synapse_type="GABAA",
                                   internal_delay="5.0",
```

```
                              weight="100.0",
                              threshold="-20.0" )
        syn_pro.tail="\n"
        if len(vs)>50:
            synR=random.sample(range(1,len(vs)), 50)
            new_vs=[]
            for s in range(50):
                new_vs.append(vs[synR[s]])
            vs=new_vs
        cons = ET.SubElement(pro, "connections", size="%d"%(len(vs)))
        cons.tail="\n"
        for i in range(len(vs)):
            ran1=float(random.random())
            ran2=float(random.random())
            con=ET.SubElement(cons, "connection",
                              id="%d"%i,
                              pre_cell_id="0",
                              pre_segment_id="%d"%vs[i][0],
                              pre_fraction_along="%f"%ran1,
                              post_cell_id="0",
                              post_segment_id="%d"%vs[i][1],
                              post_fraction_along="%f"%ran2)
            con.tail="\n"

tree = ET.ElementTree(root)
tree.write("Syn.xml")
```
