# Supplementary material for: NeuroConstruct-based implementation of structured-light stimulated retinal circuitry
Source: BMC Neurosci. 2020 Jun 24;21:28. doi: 10.1186/s12868-020-00578-0 (PMC7315481; doi:10.1186/s12868-020-00578-0)
Supplement: Supplementary file 5 — Additional file 5. Code example 4: Expanding light stimulation. [file 12868_2020_578_MOESM5_ESM.pdf]

```python
# -*- coding: utf-8 -*-
"""
Created on Wed Jan 30 22:09:33 2019
"""

import xml.etree.cElementTree as ET
import xml.etree.ElementTree as xml
from Functions import analyse

# Code that creates the XML file of the collapsing stream
if __name__ == '__main__':
    fn = 'AmacrineAA.cell.nml'
    g1,g2,g3,g4,g5,g6,g7,g8,g9,g10 = analyse(fn)

    import random
    #randomize from each group
    seg_1=random.sample(range(1,len(g1)), 10)
    seg_2=random.sample(range(1,len(g2)), 10)
    seg_3=random.sample(range(1,len(g3)), 10)
    seg_4=random.sample(range(1,len(g4)), 10)
    seg_5=random.sample(range(1,len(g5)), 10)
    seg_6=random.sample(range(1,len(g6)), 10)
    seg_7=random.sample(range(1,len(g7)), 10)
    seg_8=random.sample(range(1,len(g8)), 10)
    seg_9=random.sample(range(1,len(g9)), 10)
    seg_10=random.sample(range(1,len(g10)), 10)

    #create a list with 10 segments in each
    segArray_list=[[],[],[],[],[],[],[],[],[],[]]
    for s in range(10):
        segArray_list[0].append(g1[seg_1[s]])
        segArray_list[1].append(g2[seg_2[s]])
        segArray_list[2].append(g3[seg_3[s]])
        segArray_list[3].append(g4[seg_4[s]])
        segArray_list[4].append(g5[seg_5[s]])
        segArray_list[5].append(g6[seg_6[s]])
        segArray_list[6].append(g7[seg_7[s]])
        segArray_list[7].append(g8[seg_8[s]])
        segArray_list[8].append(g9[seg_9[s]])
        segArray_list[9].append(g10[seg_10[s]])
```

```python
root = ET.Element("inputs")
root.tail="\n"

stims=[1,2,3,4,5,6,7,8,9,10]
delays=[0,30,60,90,120,128,136,144,152,160]
dur=[30,30,30,30,8,8,8,8,8,8]

for i in range(10):
    inputs= ET.SubElement(root,"input",
                          name="stim_out_%s"%(stims[i]))
    inputs.tail="\n"
    pulse= ET.SubElement(inputs, "pulse_input",
                         delay="%s"%delays[i],
                         duration="%s"%dur[i],
                         amplitude="9.0E-5" )
    pulse.tail="\n"
    target = ET.SubElement(inputs, "target",
                           population="CellGroup_4" )
    target.tail="\n"
    sites=ET.SubElement(target, "sites", size="10")
    sites.tail="\n"
    for seg_id in segArray_list[(9-i)]:
        ss=ET.SubElement(sites, "site",
                         cell_id="0",
                         segment_id="%s"%seg_id,
                         fraction_along="0.5")
        ss.tail="\n"


tree = ET.ElementTree(root)
tree.write("Stim_Out.xml")
```
