# Supplementary material for: NeuroConstruct-based implementation of structured-light stimulated retinal circuitry
Source: BMC Neurosci. 2020 Jun 24;21:28. doi: 10.1186/s12868-020-00578-0 (PMC7315481; doi:10.1186/s12868-020-00578-0)
Supplement: Supplementary file 6 — Additional file 6. Code example 4: General functions. [file 12868_2020_578_MOESM6_ESM.pdf]

```python
import neuroml.loaders as loaders
import numpy as np
import random

# A function that returns the segments in which we create synapses
def distance(cell_1fn,c1,cell_2fn,c2,dMin):
    cell_1 = coord(cell_1fn,c1)
    cell_2 = coord(cell_2fn,c2)
    syn=[]
    for idA,a1 in cell_1.items():
        for idB,b1 in cell_2.items():
            a=np.asarray(a1)
            b=np.asarray(b1)
            #cosine similarity
            dot = np.dot(a, b)
            norma = np.linalg.norm(a)
            normb = np.linalg.norm(b)
            cos = dot / (norma * normb)
            #distance between vectors
            dis=np.linalg.norm(a-b)
            #probability
            #sim=1/cos
            ran=float(random.random())
            if dis<dMin and ran<abs(cos):
                syn.append([idA,idB])
    return(syn)

def coord(cell_file,v):
    vectors={}
    doc = loaders.NeuroMLLoader.load(cell_file)
    cell = doc.cells[0]
    segmentset=cell.morphology.segments
    for s in segmentset:
        for g in [ s.proximal]:
            if g is None:
                coord_pro = [v[0]+par.distal.x, v[1]+par.distal.y]
            else:
                coord_pro = [v[0]+g.x, v[1]+g.y]
        for c in [ s.distal]:
```

```
            if c is None:
                continue
            coord_dis = [v[0]+c.x, v[1]+c.y, v[2]+c.z]
        par = s
        vectors[s.id] = coord_dis
    return vectors

def analyse(cell_file):

    doc = loaders.NeuroMLLoader.load(cell_file)
    print("Loaded morphology file from: "+cell_file)

    cell = doc.cells[0]
    morph = cell.morphology
    segs_all = []
    seg_len_d = {}
    segs=[]

    cell.summary()
    cables = 0
    for sg in morph.segment_groups:
        segs = cell.get_ordered_segments_in_groups(sg.id)[sg.id]

        cable = sg.neuro_lex_id=="sao864921383"
        if cable:
            cables+=1

        if len(segs)>2:
            for i in range(len(segs)):
                if not(segs[i].id in segs_all):
                    segs_all.append(segs[i].id)

    print("%s NEURON sections/cables found"%cables)

    _, _, length_to_distal = cell.get_ordered_segments_in_groups("all",
                                  include_path_lengths=True)

    seg_len_d = length_to_distal['all']
```

```
g1, g2, g3, g4, g5, g6, g7, g8, g9, g10 = [], [], [], [], [], [], [], [], [], []

for key in segs_all:
    if seg_len_d[key]<17.556:
        g1.append(key)
    elif seg_len_d[key]<35.112:
        g2.append(key)
    elif seg_len_d[key]<55.668:
        g3.append(key)
    elif seg_len_d[key]<70.224:
        g4.append(key)
    elif seg_len_d[key]<87.781:
        g5.append(key)
    elif seg_len_d[key]<105.337:
        g6.append(key)
    elif seg_len_d[key]<122.893:
        g7.append(key)
    elif seg_len_d[key]<140.449:
        g8.append(key)
    elif seg_len_d[key]<158.005:
        g9.append(key)
    elif seg_len_d[key]<175.561:
        g10.append(key)

return g1,g2,g3,g4,g5,g6,g7,g8,g9,g10
```
